# Effect of the consonant context on the corner vowel produced by native and Chinese speakers: based on AESOP corpora

**DOI:** 10.3389/fpsyg.2025.1598904

**Published:** 2025-09-17

**Authors:** Hongming Zhu

**Affiliations:** School of Foreign Languages, Harbin University, Harbin, China

**Keywords:** formant F1 and F2, Chinese, English pronunciation, AESOP ILAS 1, vowel space, consonant context

## Abstract

This study investigated the differences in the consonant context on the four corner vowels (/i/, /u/, /æ/, and /ɑ/) between native and Chinese speakers using the AESOP-ILAS 1 corpus dataset. Ninety-six test subjects with manually adjusted data comprising of 12 native speakers (the control group) and 84 non-native speakers (the targeted group), with 58 Chinese speakers (even gender distribution), 22 Taiwanese speakers (quasi-equal gender distribution), and 4 Hakka-speaking women, were chosen for the analysis. By adopting continuous speech initially, a general comparison of the vowel space for the native and the non-native speakers is presented. Next, the vowel space of the liquid and glide sonorants in the onset and the coda position are compared. The nasal sonorants are compared in their subsections. Finally, the study analyzed the obstructive sounds and compared the vowel spaces of different pronunciation sites (alveolar, labial, posterior alveolar, and palatal sounds). Since male speakers typically having longer vocal tracts, their vowels may be more centralized or lower in pronunciation compared to females. In order to comprehensively understand the spatial distribution of vowels, it is valuable to further analyze whether there are differences in vowel production patterns between male and female speakers in the native and non-native language groups. Compared to native speakers, Chinese English learners have a larger range of vowel spaces, which may be due to the fact that the corpus is collected from sentences rather than isolated words. Chinese learners exhibit lower F1 and F2 values on corner vowels, with particularly significant differences between /ɑ/ and /u/. These differences are influenced by adjacent phonemes, such as the/w/sound at the beginning and end of syllables. The study suggest that improving the stress distribution of Chinese learners in sentences will significantly enhance their pronunciation level.

## 1 Introduction

Vowels play a crucial role in English pronunciation, especially in Second Language Acquisition (SLA; Celce-Murcia et al., 1996). Pronunciation issues often arise for Chinese speakers (L1) learning English, especially when producing English vowels (Flege et al., 1997). Although existing acoustic research has focused on these phonetic challenges, most of them are concentrated in controlled experimental environments, making it difficult to reflect real language usage scenarios. As Chen et al. (2001) pointed out, the vowel space of second language learners is usually more constrained than that of native speakers, which means that their range of vowel sounds and perceptual abilities are relatively limited.

Standard American English is typically described as having around 10–12 distinct vowels (Peterson and Barney, 1952; Ladefoged and Johnson, 2015), which are categorized based on tongue position: front, central, or back. Four key corner vowels—/i/ (high-front), /u/ (high-back), /æ/ (low-front), and /ɑ/ (low-back)—establish the vowel space for a speaker, varying acoustically due to differences in vocal tract anatomy and individual articulation patterns (Hillenbrand et al., 1995). Research in second language (L2) acquisition has shown that the production of these vowels is influenced by multiple factors, including age of acquisition (Flege et al., 1999), first language (L1) phonological transfer (Best and Tyler, 2007), and phonetic training experience (Bradlow et al., 1997). For Chinese English learners, their pronunciation difficulties are not only due to differences in the phoneme system between their native language and the target language (Flege, 1995), but also involve fundamental differences in the acoustic implementation of similar vowels. Although the basic vowel system proposed by Jones (1960) still has reference value, contemporary research emphasizes the reshaping effect of perceptual training and pronunciation learning on the production of second language vowels, especially for phonemes that do not exist in the native language phonology (Escudero, 2005; Munro and Derwing, 2008).

Cardinal vowels serve as reference points for vowel articulation, with each language's vowels mapped relative to these idealized positions. However, the exact proximity of these vowels in both English and Chinese may differ. For example, the/i/sound in “heed” is closer to cardinal vowel 1, which may not align perfectly with the Chinese /i/ in “衣” (meaning: clothe). This discrepancy highlights the challenges faced by Chinese learners, who may produce vowels with significantly different acoustic properties from native speakers, even when articulating the same sounds. Understanding these acoustic differences is essential for improving pronunciation accuracy.

Although early research focused mainly on experimental data, there is still a research gap regarding the accuracy of English pronunciation by Chinese learners, especially in terms of pronunciation performance in natural language streams. The acoustic and pronunciation characteristics of vowels are influenced by multiple linguistic factors, including the phonological environment, speed, and production context of vowels. Among them, adjacent consonants (especially consonants before and after the target vowel) will significantly alter their spectral characteristics. For instance, Stevens and House (1963) demonstrated that consonant context can significantly alter the formant values of vowels, with F2 shifts varying based on the surrounding consonants. Further research by Hillenbrand et al. (2001) confirmed the influence of phonetic environments on vowel identification, though the role of consonants in vowel intelligibility is less pronounced.

Given these findings, it is crucial to examine the acoustic differences in vowel production between native and non-native speakers. This study focuses on the pronunciation of the four corner vowels (/i/, /u/, /æ/, and /ɑ/) by 488 Taiwanese Chinese (L1) speakers from the AESOP-ILAS (Asian English Speech Corpus Project) dataset. Specifically, the study analyzes first formant (F1) and second formant (F2) frequencies of these vowels produced by 96 test subjects, including 12 native English speakers (the control group) and 84 non-native speakers (the target group). The results are compared across different consonant contexts, including sonorants, nasals, and obstruents, as well as the place of articulation (alveolar, labial, post-alveolar, and velar) to better understand the effects of consonantal environment on vowel production.

The research questions of this study are as follows:

1. Compared with the vowels produced by American native English speakers, how different are the formant F1 and F2 values produced by Chinese learners?
2. How does the vowel space differ in size and shape for the Chinese learners as compared with the Americans?
3. To what extent does the phonological environment influence the production of the target monophthongs?

## 2 Methodology

### 2.1 Data description

This study is based on the AESOP-ILAS (Asian English Speech Corpus Project—Institute of Linguistics, Academia Sinica) corpus, which is composed of the speech of Taiwanese learners of English. The data is separated into two parts: AESOP-ILAS 1 and AESOP-ILAS 2. The AESOP-ILAS 1 corpus data is adopted in this study as it contains a more diverse dataset. This enables better separation of segmental and suprasegmental characteristics. AESOP-ILAS 1 is 8.64 GB in size and has 500 hours of of speech recordings, including L1 English speech data by 12 American English native speakers and L2 English speech by 488 Taiwanese Chinese speakers coming from 12 universities or institutes located in Taiwan. The recording time of each speaker is approximately 1 hour. The recording time of each L1 speaker is approximately 5.25 and 8.7 hours for each L2 speaker.

### 2.2 Test subjects

The dataset has 12 native English speakers and 488 non-native speakers. For the native speakers, the dataset is evenly distributed with six males and six females. As for the non-native speakers, 58 spoke Chinese, with a nearly even distribution between men and women; 22 spoke Taiwanese, with a quasi-equal number of men and women; and four women spoke Hakka. Since nearly 70% of the non-native speakers are Chinese speakers, the background language will not influence the vowel production. Most of the non-native men, 15 in total, had between 5 and 10 years of English experience; 8 had between 10 and 15 years of experience; 6 had between 15 and 20 years of experience; and 1 had more than 20 years of experience. As for women, 13 had between 5 and 10 years of experience, 13 had between 10 and 15 years of experience, and 2 had between 15 and 20 years of experience.

All recordings were made in a quiet room such as a classroom or in the office of the instructor, using a Sennheiser PC 155 headset with a unidirectional microphone. The capture and digitalization were made with the TWNAESOP Recording software from the CUHK-SIAT (the Chinese University of Hong Kong and Shenzhen Institutes of Advanced Technology). The labels were automatically generated using the Hidden Markov Toolkit and some of them were manually adjusted.

Among the 500 subjects in the AESOP1 database, 96 had manually adjusted data, comprising of 12 native speakers, who are referred to as the control group, and 84 non-native speakers, who are referred to as the targeted group. Each of the 96 test subjects was asked to finish seven reading tasks and one picture description task. With the exception of the last picture description task, all of the seven reading tasks have been transcribed. Consequently, our four target corner vowels /i/, /u/, /æ/, and /ɑ/ are extracted from the sentences used in these seven tasks.

The recording of AESOP-ILAS 1 was conducted from 2009 to 2012 and it contains eight recorded tasks comprising six elicited read speech tasks, one fully aided computer-prompted dialogue task, and one partially aided picture description task. All of the transcriptions of the recordings are presented in Appendices A–G, where an asterisk (^*^) indicates that there are no corner vowels appear in that sentence.

### 2.3 Formant measurements

The four targeted corner vowels are compared based on the averaged formant (F1 and F2) values from the control group (L1) with that of the target group (L2). Instead of comparing the formants (F1 and F2) at the middle of the target vowel as in Chen (1999), this study proposes to measure them at three locations: the start, middle, and end portion of the vowel and then take their average. These targeted corner vowels were obtained from the sentences in tasks 1–7. For example, the /ɑ/ from the word “Apartment” [əpɑrtmənt], “supermarket” [supərmɑrkɪt], and so on. From the dataset, 4,982 tokens for the /ɑ/ sound, 7,753 tokens for the /æ/ sound, 11,803 tokens for the /i/ sound, and 6,806 tokens for the /u/ sound were extracted. From these tokens, the relevant formant values are extracted.

**Figure 1:**
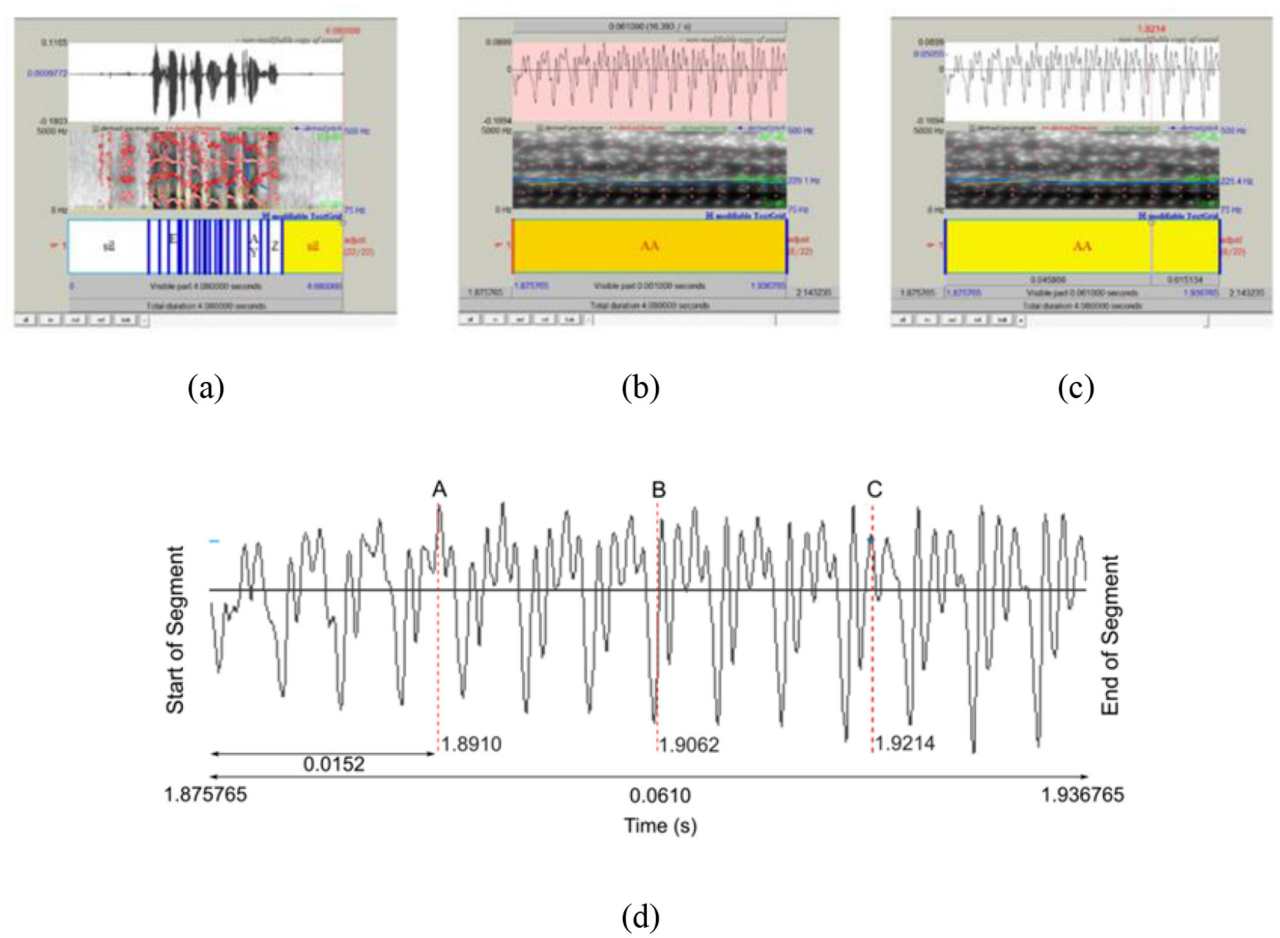
A visualization of capturing a vowel. **(a)** Visual representation of the sound making up the speech; **(b)** selection of the desired vowel, /ɑ/, sound; **(c)** extraction of the formants F1 and F2 at a particular point in time; **(d)** extraction of Formant Frequencies.

**Figure 2:**
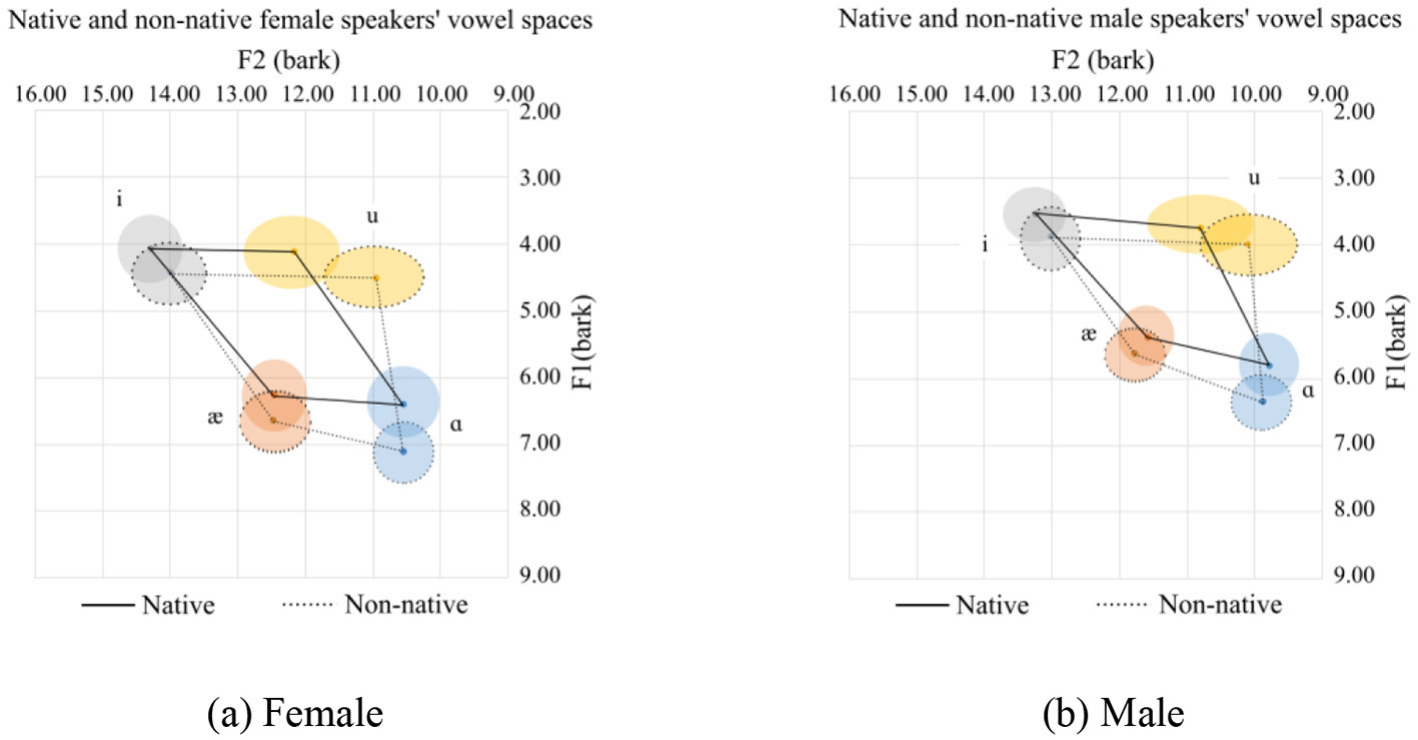
Vowel space for native and Chinese speakers. **(a)** Female. **(b)** Male.

The procedure is based on using Praat Software, version 6.2.19, running on a Microsoft Windows PC. It is as follows: for a given speaker, the audio sample file for the task was loaded together with a labeled file containing the time segments of each phoneme. This allowed the localization of the desired corner vowel in the audio segment, as shown in Figure 1, where the top window shows the waveform, the middle window shows the spectrogram, and the lower window shows the time intervals of the occurrence of the phonemes. Once such a segment is identified, in this case, /ɑ/, from the sentence “I said apartment five times,” which begins at 1.875765 second (s) and ends at 1.936765 s, the time duration (0.0610 s) is sliced into four equal intervals of 0.0152 s (0.0610/4) to obtain three sample points (A at 1.8910 s, B at 1.9062 s, and C at 1.9214 s) as shown in Figure 2. Using linear predictive coding (LPC), the formant (F1 and F2) values at these three locations are computed by the software based on the spectra. These three pairs of formant values are then averaged to produce a single pair of formant (F1 and F2) values for that particular corner vowel. This average value gives a more representative value as it takes into consideration the transition (high to low) at the start of the vowel, the steady state, and the final transition (high to low) at the end of the phoneme. By so doing, any hesitation or on-the-fly correction in the pronunciation of the corner vowel can be detected. This procedure is repeated for any further occurrences of that particular vowel in the speech segment.

The same set of procedures is carried out again for the other corner vowels of interest and is repeated for all the participants. With this, the core data set of this study is obtained and it contains 31,344 distinct data points.

## 3 Results and discussion

### 3.1 General view of the corner vowels production for native and Chinese speakers

The vowel space for native and Chinese females is shown in Figure 2a in the bark scale. Since the tongue height is inversely related to the F1, the lower the value, the higher the tongue position. Regarding the F2, the higher the value, the more fronted the tongue position. In this regard, as compared with their native counterparts, Chinese female speakers tend to use a lower and more pulled-back tongue position relative to the palate. This difference in the height of the tongue is nearly similar for the high vowels (/i/ and /u/) and slightly smaller for the lower front vowels (/æ/). As for the lower back vowel, /ɑ/, however, is pronounced at a much lower tongue position. As for the advancement of the tongue, which can be interpreted from the F2 value, the high vowels are pronounced with a more pulled-back tongue position. The pull-back is more for /u/ than for the high vowels. As for the low vowels (/æ/ and /ɑ/), they are pronounced with the same advancement of the tongue.

The following observations can be made about the Chinese speakers:

1. For /i/, Chinese speakers tend to pronounce it with a lower, more open lip and a more pulled-back tongue position.
2. For /u/, Chinese speakers tend to pronounce it with a lower and more pulled-back tongue position than their native counterparts. Additionally, there are more variations in the F2 for /u/ in native and Chinese speakers than for the other corner vowels.
3. For /æ/, the tongue position is lower with a more open mouth position.
4. For /ɑ/, the tongue is in a lower position with more rounded lips.

The mean and standard deviations of the formants F1 and F2 in hertz, together with the number of speakers and tokens collected for the females and males, are summarized in Table 1. For the males, a similar observation to the females can be observed.

**Table 1:** F1 and F2 for native and Chinese speakers.

		**Native**								**Chinese**							
Female	C. Vowel	ɑ		æ		i		u		ɑ		æ		i		u	
	Formant	F1	F2	F1	F2	F1	F2	F1	F2	F1	F2	F1	F2	F1	F2	F1	F2
	Mean	690	1414	672	1,888	405	2,478	412	1,832	787	1,407	720	1,896	444	2,385	453	1,520
	SD	153	264	154	282	1,235	311	146	395	137	217	125	291	119	360	118	349
	#Speakers	6		6		6		6		44		44		44		44	
	#Tokens	310		486		753		423		2,288		3,545		5,380		3,125	
P.value	ɑ	F1: p.value: 0.000 Cohen's d: −0.695; F2: p.value: 0.000 Cohen's d: 0.032															
	æ	F1: p.value: 0.000 Cohen's d: −0.037; F2: p.value: 0.000 Cohen's d: −0.027															
	i	F1: p.value: 0.000 Cohen's d: −0.087; F2: p.value: 0.000 Cohen's d: 0.26															
	u	F1: p.value: 0.000 Cohen's d: −0.336; F2: p.value: 0.000 Cohen's d: 0.879															
Male	C. Vowel	ɑ		æ		i		u		ɑ		æ		i		u	
	Formant	F1	F2	F1	F2	F1	F2	F1	F2	F1	F2	F1	F2	F1	F2	F1	F2
	Mean	609	1,244	555	1,646	346	2,121	369	1,484	678	1,264	585	1,698	383	2,051	396	1,327
	SD	118	179	110	190	101	262	110	349	107	188	97	213	121	244	119	308
	#Speakers	6		6		6		6		40		40		40		40	
	#Tokens	313		486		754		426		2,071		3,236		4,916		2,834	
P.value	ɑ	F1: p.value: 0.000 Cohen's d: −0.63; F2: p.value: 0.066 Cohen's d: −0.10															
	æ	F1: p.value: 0.000 Cohen's d: −0.30; F2: p.value: 0.000 Cohen's d: −0.24															
	i	F1: p.value: 0.000 Cohen's d: −0.31; F2: p.value: 0.000 Cohen's d: 0.28															
	u	F1: p.value: 0.000 Cohen's d: 0.10; F2: p.value: 0.000 Cohen's d: 0.50															

Out of the 849 tokens with the /u/ sound by the native speakers, 287 tokens begin with the /t/ sound, and 334 tokens begin with the /j/ sound. Among the 849 tokens for the /u/ sound in the coda position, only 24 tokens are followed by a silent sound, and an /l/ or /s/ sound follows the rest. Vowels like /i/, /u/, and /ɑ/ are mostly used without a coda, and usually, the consonant sounds /l/ and /s/ are on the onset position of the following word. For example, the /l/ sound comes from the word “learn” in the sentence: “if you want to learn Vietnamese, I think it will be easier than Japanese” (Appendix C Record 2013). Among the 287 tokens that begin with the T sound, it is quite possible that the carrier word is the preposition word “to.” Similarly, among the 334 tokens that begin with a /j/ sound, the majority are for the word “you,” and few are for “January” (36 tokens), “computer” (11 tokens), “usually” (12 tokens), and “disputing” (12 tokens). The native speakers pronounce the vowel correctly by not stressing the preposition “to”; consequently, they pronounce it as a reduced vowel like a schwa. This effect can be observed in the following sentences: Did he go to the hospital? (Appendix C Record 2001), He had no trouble learning how to make a video (Appendix C Record 2011), and If you want to learn Vietnamese (Appendix C Record 2013). This is also done for words in the infinitive form. As for the 263 tokens with the pronunciation of “you,” out of the 344 tokens with the Y sound, only 12 “you's” should be emphasized according to the text: I can run faster than you can. Expect for this sentence, all the 249 other “you's” should not be emphasized. The native speakers know that when “you” is used as a pronoun or as a functional word, it must not be emphasized.

When used as a function word, both “to” and “you” are pronounced with reduced strength by the native speakers, resulting in the /u/ sounding like schwa, which will put the tongue in a much more fronted position. In contrast, the Chinese speakers put the same stress on the syllables when pronouncing “to” and “you,” which pulls back and lowers the tongue to pronounce the/u/sound.

The pronunciation of the /u/ sound in the following subsections will likely follow the same general pattern.

### 3.2 Effect of the consonant context on the corner vowel

#### 3.2.1 Sonorants

Sonorants are a group of speech sounds produced with a relatively open vocal tract, allowing air to flow freely. These sounds include glide, nasals, and liquids. Since most vowels are produced with the consonants in the onset or coda positions, the consonantal effects on the vowels will be examined. The sonorants have been split into the liquids and the nasals. The nasals are treated separately because the vowels are significantly affected by nasalization. These create anti-formants, which reduce the intensity of the formants, blurring the peak of formant frequencies, which results in an erroneous calculation in formant tracking.

##### 3.2.1.1 Sonorants in the onset position

**Figure 3:**
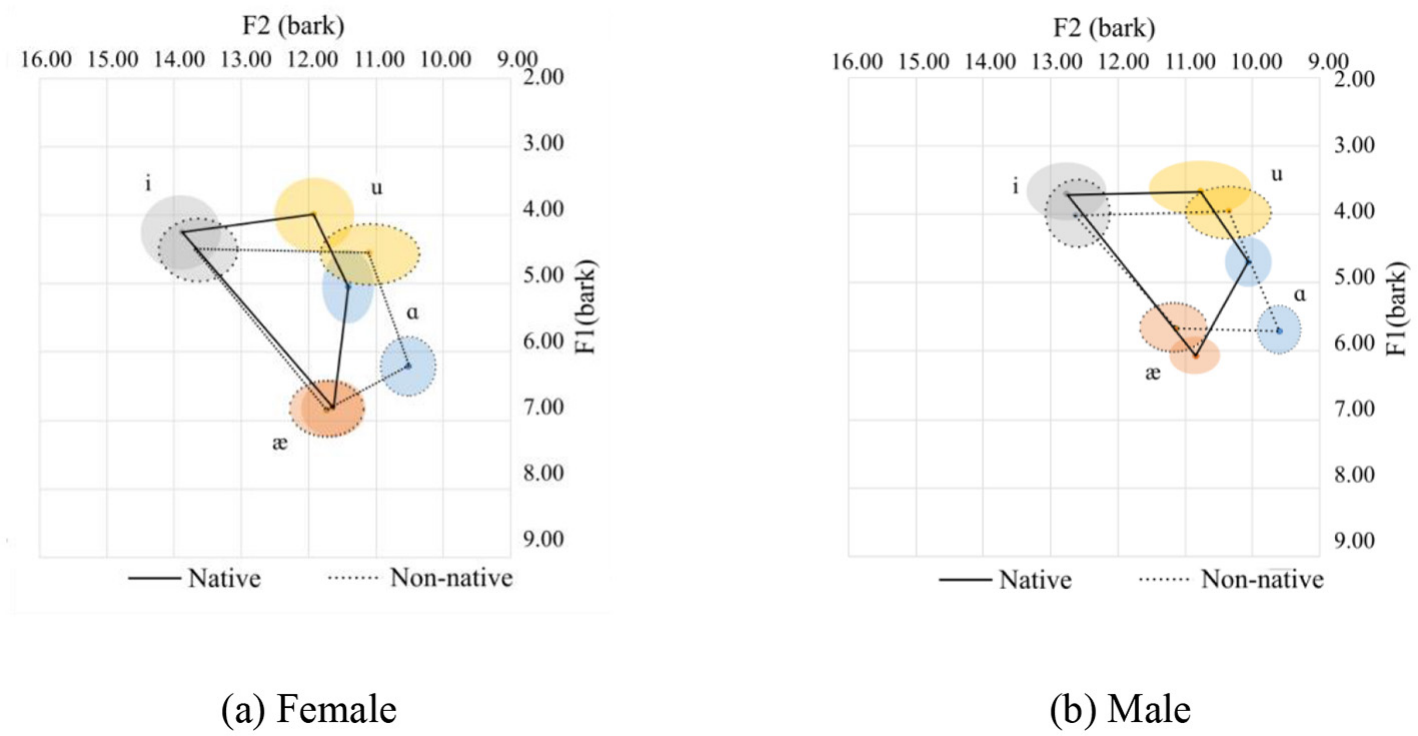
Vowel spaces for onset sonorants. **(a)** Female. **(b)** Male.

In this case, the liquid consonants /r/ and /l/ and the glide consonants /j/ and /w/ were included while the nasal ones were excluded. It can be seen from the Figures 3a, b that besides the /ɑ/ sound, the other three corner vowels /i/, /æ/, and /u/, almost follow the general trend. The statistics of the formants are given in Table 2. One possible explanation for the deviation in the pronunciation of /ɑ/ is as follows:

**Table 2:** The F1 and F2 by native and Chinese speakers for onset sonorants.

		**Native**								**Chinese**							
Female	C. Vowel	ɑ		æ		i		u		ɑ		æ		i		u	
	Formant	F1	F2	F1	F2	F1	F2	F1	F2	F1	F2	F1	F2	F1	F2	F1	F2
	Mean	518	1,606	741	1,668	426	2,346	394	1,773	661	1,399	746	1,700	452	2,275	455	1,547
	SD	135	191	117	261	137	363	102	419	119	186	118	296	121	372	100	318
	#Speakers	6		6		6		6		44		44		44		44	
	#Tokens	53		66		180		184		401		472		1,315		1,355	
P.value	ɑ	F1: p.value: 0.000 Cohen's d: −1.18; F2: p.value: 0.000 Cohen's d: 1.10															
	æ	F1: p.value: 0.751 Cohen's d: −0.04; F2: p.value: 0.401 Cohen's d: −0.10															
	i	F1: p.value: 0.001 Cohen's d: −0.21; F2: p.value: 0.018 Cohen's d: 0.19															
	u	F1: p.value: 0.000 Cohen's d: −0.60; F2: p.value: 0.000 Cohen's d: 0.68															
Male	C. Vowel	ɑ		æ		i		u		ɑ		æ		i		u	
	Formant	F1	F2	F1	F2	F1	F2	F1	F2	F1	F2	F1	F2	F1	F2	F1	F2
	Mean	473	1,296	639	1,469	363	1,984	359	1,480	594	1,205	590	1,544	399	1,939	390	1,376
	SD	88	139	85	180	104	311	106	354	90	132	92	235	127	270	101	280
	#Speakers	6		6		6		6		40		40		40		40	
	#Tokens	56		66		180		186		360		437		1,192		1,231	
P.value	ɑ	F1: p.value: 0.000 Cohen's d: −1.34; F2: p.value: 0.000 Cohen's d: 0.68															
	æ	F1: p.value: 0.000 Cohen's d: 0.53; F2: p.value: 0.000 Cohen's d: −0.32															
	i	F1: p.value: 0.000 Cohen's d: −0.28; F2: p.value: 0.049 Cohen's d: 0.16															
	u	F1: p.value: 0.000 Cohen's d: −0.30; F2: p.value: 0.000 Cohen's d: 0.35															

Among the four sonorants (/r/, /l/, /j/, /w/) on the onset position of the /ɑ/ sound, only the /w/ sound appears in this case. The /w/ sound in the onset position can affect the production of the /ɑ/ sound as /w/ is a labial-velar sound, which involves rounding the lips and raising the tongue, similar to the pronunciation of velars. When /w/ is followed by an /ɑ/, which is a low vowel, the lips tend to remain rounded, meanwhile, the tongue's elevation is still at a velar sound position, resulting in a more rounded or protruded lip position for the /ɑ/ sound. This tongue position results in lower F1 and F2 values. We can also see from the statistics that the pronunciation of the corner variables /æ/ and /i/ are relatively similar among the native and non-native females. As for the males, only the F2 for /i/ is relatively similar.

##### 3.2.1.2 Sonorant in the coda position

The liquid consonants /r/ (12 tokens) and /l/ (86 tokens) and the glide consonants /j/ (48 tokens) and /w/ (87 tokens) were considered while the nasal sounds were excluded. The formant F1 and F2 values in Hertz are given in Table 3.

**Table 3:** The F1 and F2 by native and Chinese speakers for coda sonorants.

		**Native**								**Chinese**							
Female	C. Vowel	ɑ		æ		i		u		ɑ		æ		i		u	
	Formant	F1	F2	F1	F2	F1	F2	F1	F2	F1	F2	F1	F2	F1	F2	F1	F2
	Mean	712	1,288	822	1,640	396	2,033	396	1,792	836	1,307	756	1,812	429	2,084	440	1,466
	SD	109	206	136	169	72	429	112	437	111	164	110	260	76	424	75	370
	#Speakers	6		6		6		6		44		44		44		44	
	#Tokens	132		30		64		115		969		220		475		827	
P.value	ɑ	F1: p.value: 0.000 Cohen's d: −1.11; F2: p.value: 0.231 Cohen's d: −0.11															
	æ	F1: p.value: 0.000 Cohen's d: 0.58; F2: p.value: 0.000 Cohen's d: −0.68															
	i	F1: p.value: 0.000 Cohen's d: −0.43; F2: p.value: 0.363 Cohen's d: −0.12															
	u	F1: p.value: 0.000 Cohen's d: −0.54; F2: p.value: 0.000 Cohen's d: 0.86															
Male	C. Vowel	ɑ		æ		i		u		ɑ		æ		i		u	
	Formant	F1	F2	F1	F2	F1	F2	F1	F2	F1	F2	F1	F2	F1	F2	F1	F2
	Mean	620	1,136	672	1,423	358	1,701	350	1,488	717	1,191	603	1,671	360	1,827	366	1,297
	SD	69	121	76	119	73	360	56	381	83	143	85	203	68	329	57	313
	#Speakers	6		6		6		6		40		40		40		40	
	#Tokens	132		30		62		118		881		200		424		747	
P.value	ɑ	F1: p.value: 0.000 Cohen's d: −1.19; F2: p.value: 0.000 Cohen's d: −0.39															
	æ	F1: p.value: 0.000 Cohen's d: 0.82; F2: p.value: 0.000 Cohen's d: −1.27															
	i	F1: p.value: 0.788 Cohen's d: −0.02; F2: p.value: 0.000 Cohen's d: −0.37															
	u	F1: p.value: 0.000 Cohen's d: −0.28; F2: p.value: 0.000 Cohen's d: 0.59															

**Figure 4:**
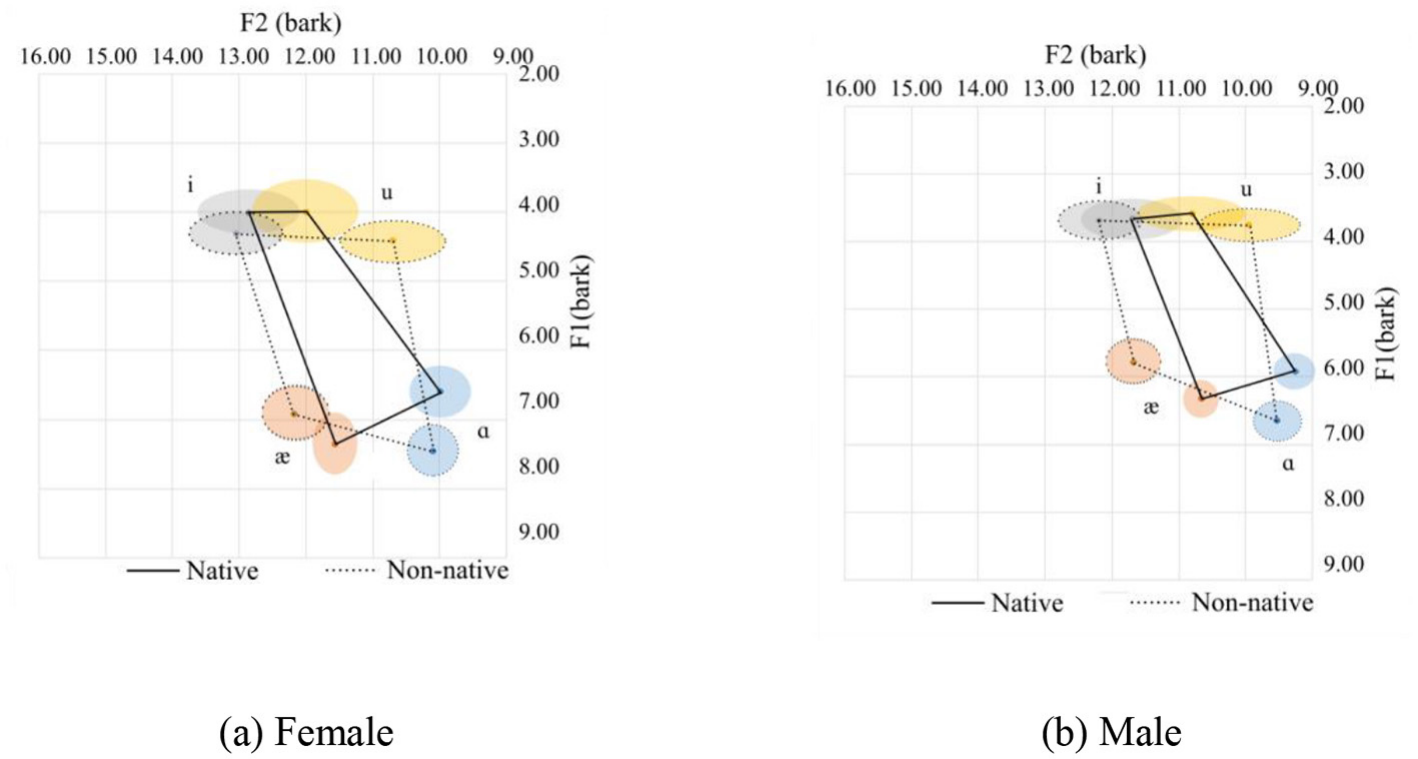
Vowel spaces for coda sonorants. **(a)** Female. **(b)** Male.

In general, Chinese female speakers tend to pronounce the corner vowels with a more frontal tongue position at a lower tongue height, except for /æ/, which is pronounced at a slightly higher position. Additionally, the vowel space for the Chinese speakers is larger than that of the native speakers, and the distance between /i/ and /u/ for the native speakers is shorter than their Chinese counterparts. This is primarily due to the pronunciation of /i/. Of the 126 tokens from native speakers for male and female, the majority (102 tokes) end with sonorants /w/, and /l/ and /r/ sounds are 12 tokens for each. The /w/ sound in the coda position can affect the production of the /i/ sound as /w/ is a labial-velar sound, which involves rounding the lips and raising the tongue, similar to the pronunciation of velars. When /i/ is followed by a /w/ sound, which is a low vowel, the lips tend to be prepared for a rounded position. Meanwhile, the tongue's position is relatively posterior, resulting in a lower F1 and F2 value. The tongue position can be visualized in Figures 4a, b for the females and males respectively. The statistics are provided in Table 4, where it can be seen that there is a small Cohen's d value for F2 for /ɑ/ and for /i/ for the females and F1 for /i/ for the males.

#### 3.2.2 Nasals

Nasal sounds are speech sounds produced by allowing air to flow through the nose as well as the mouth. The pronunciation is achieved by lowering the soft palate (or velum) at the back of the mouth, allowing air to pass through the nasal cavity while also passing through the mouth. The resulting sound is often described as having a nasal quality. The nasalization of the sound produced can significantly affect the formant F1 and F2 values due to the formation of anti-formants. Hence the nasal sonorants are considered here.

##### 3.2.2.1 Nasals in the onset position

In this case, the consonants /n/, /m/, and /η/ were included. In general, Chinese speakers tend to pronounce the upper corner vowels /i/ and /u/ with a more pulled-back and at a lower tongue height position. Additionally, the tongue height for /i/ and /u/ is similar. As for the low corner vowels /æ/ and /ɑ/, /æ/ is similar to the native's pronunciation, with a slightly more frontal pronunciation in the females' pronunciation, while it is different for the males' pronunciation, which is much more frontal. On the other hand, /ɑ/ is quite different as it is pronounced with a more frontal and lower tongue position than the female native speakers. The vowel space for the Chinese speakers is slightly smaller than their native counterparts for the sonorant nasal coda.

**Figure 5:**
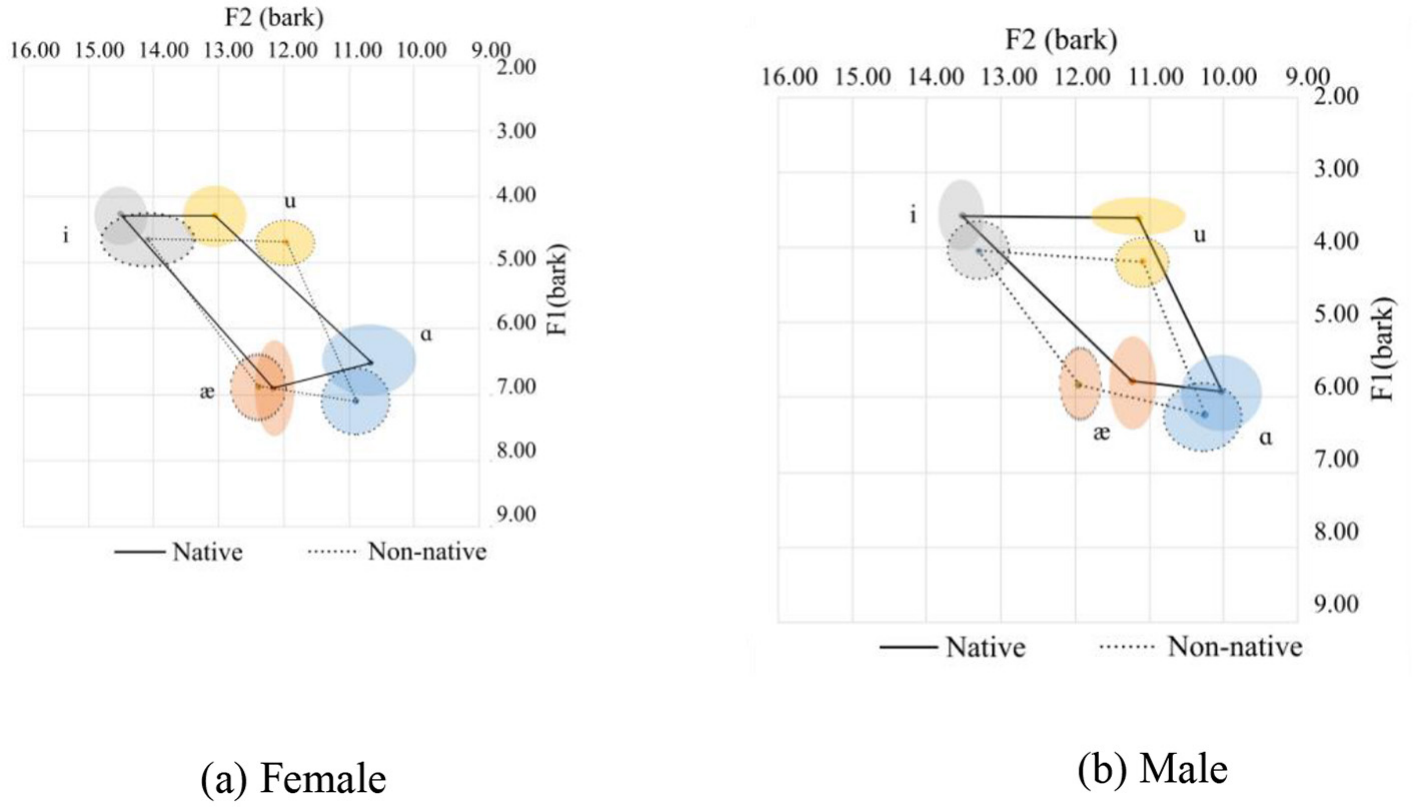
Vowel space for onset nasals. **(a)** Female. **(b)** Male.

Although the two graphs, Figures 5a, b, show an inconsistent pattern between the female and the male speakers, they follow the general pattern, as discussed in Section 3.1. The statistics are summarized in Table 4.

**Table 4:** The F1 and F2 by native and Chinese speakers for onset nasals.

		**Native**								**Chinese**							
Female	C. Vowel	ɑ		æ		i		u		ɑ		æ		i		u	
	Formant	F1	F2	F1	F2	F1	F2	F1	F2	F1	F2	F1	F2	F1	F2	F1	F2
	Mean	705	1,453	766	1,800	424	2,559	427	2,071	785	1,490	755	1,866	468	2,431	471	1,753
	SD	147	346	212	167	106	287	108	305	145	251	145	233	104	422	82	243
	#Speakers	6		6		6		6		44		44		44		44	
	#Tokens	95		18		125		30		669		107		923		218	
P.value	ɑ	F1: p.value: 0.000 Cohen's d: −0.55; F2: p.value: 0.000 Cohen's d: −0.13															
	æ	F1: p.value: 0.772 Cohen's d: 0.07; F2: p.value: 0.000 Cohen's d: −0.29															
	i	F1: p.value: 0.000 Cohen's d: −0.42; F2: p.value: 0.000 Cohen's d: 0.31															
	u	F1: p.value: 0.000 Cohen's d: −0.51; F2: p.value: 0.000 Cohen's d: 1.26															
Male	C. Vowel	ɑ		æ		i		u		ɑ		æ		i		u	
	Formant	F1	F2	F1	F2	F1	F2	F1	F2	F1	F2	F1	F2	F1	F2	F1	F2
	Mean	624	1,302	609	1,560	350	2,205	352	1,559	663	1,348	611	1,742	401	2,139	415	1,528
	SD	131	238	155	160	121	202	53	320	120	241	123	175	94	247	75	179
	#Speakers	6		6		6		6		40		40		40		40	
	#Tokens	98		18		126		30		611		114		837		198	
P.value	ɑ	F1: p.value: 0.003 Cohen's d: −0.32; F2: p.value: 0.082 Cohen's d: −0.19															
	æ	F1: p.value: 0.941 Cohen's d: −0.01; F2: p.value: 0.000 Cohen's d: −1.05															
	i	F1: p.value: 0.000 Cohen's d: −0.52; F2: p.value: 0.004 Cohen's d: 0.27															
	u	F1: p.value: 0.000 Cohen's d: −0.86; F2: p.value: 0.436 Cohen's d: 0.15															

##### 3.2.2.2 Nasals in the coda position

**Figure 6:**
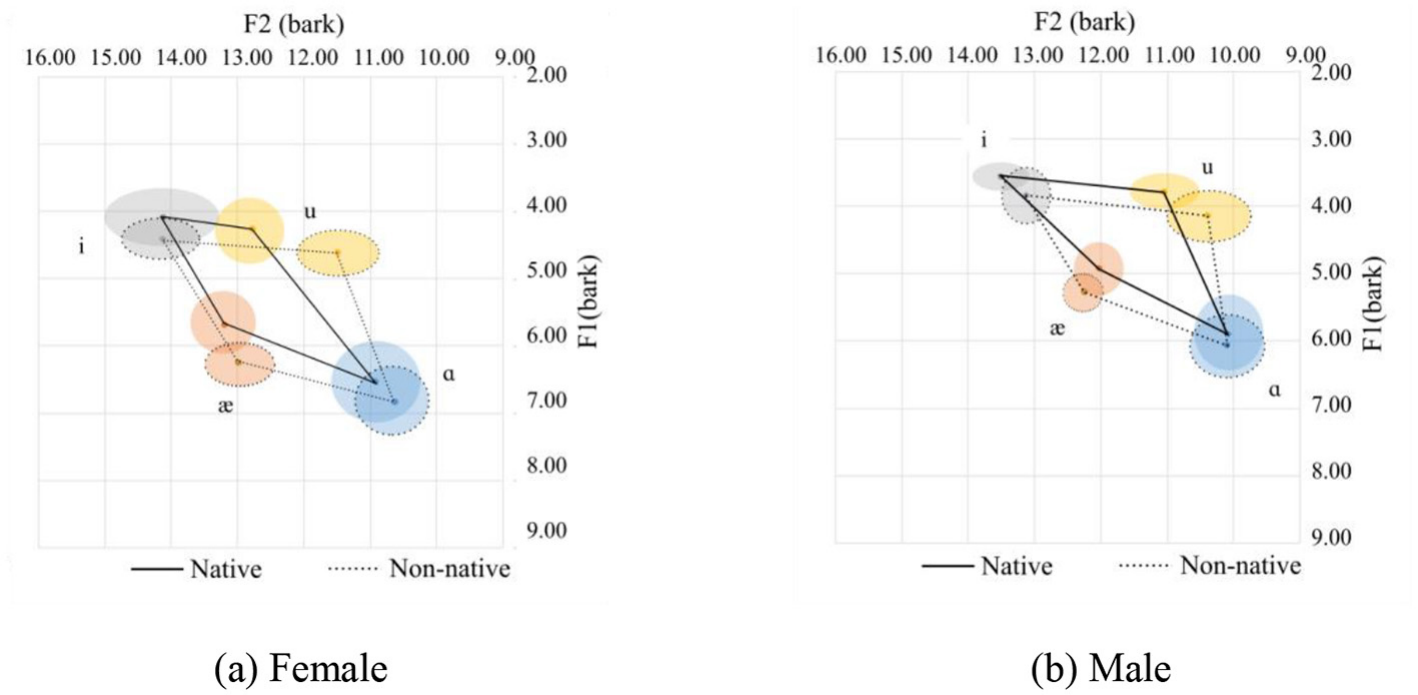
Vowel spaces for coda nasals. **(a)** Female. **(b)** Male.

It can be observed from Figures 6a, b that the vowel space for the Chinese speakers is much larger than that of the native-speakers. Except for /i/, the Chinese female speakers pronounce all the other corner vowels with a more posterior tongue. It is also observed that the tongue' elevation is lower for the Chinese speakers. /u/ is also pronounced with a much more posterior tongue position by the Chinese speakers.

The three corner vowels /i/, /u/, and /ɑ/ follow a similar pattern except for /æ/, for which the native speakers pronounce with a conspicuous higher tongue position with more rounded lips than the Chinese speakers. A possible reason is as follows:

Of the 972 tokens for the native speakers' /æ/ sound, 300 tokens end with the sonorant nasals (/n/, /m/, and /η/). Among these, 288 tokens belong to the alveolar sound /n/, and the 12 remaining tokens belong to the /η/ sound. There are no tokens for the /m/ sound. So, generally speaking, after pronouncing the /æ/ sound, the tongue will be raised in anticipation of the nasal /n/ sound, which is an alveolar nasal sound. Naturally, the tongue will move closer to the alveolar ridge area, lowering the F1 value. The statistics of the data are summarized in Table 5 where we can see the strong similarity in the pronunciation of /ɑ/ for the males.

**Table 5:** The F1 and F2 by native and Chinese speakers for coda nasals.

		**Native**								**Chinese**							
Female	C. Vowel	ɑ		æ		i		u		ɑ		æ		i		u	
	Formant	F1	F2	F1	F2	F1	F2	F1	F2	F1	F2	F1	F2	F1	F2	F1	F2
	Mean	709	1,507	594	2,110	405	2457	425	1,988	748	1,433	663	2,049	440	2,435	462	1,641
	SD	158	334	118	307	102	470	113	304	145	270	93	296	73	373	81	306
	#Speakers	6		6		6		6		44		44		44		44	
	#Tokens	78		150		46		42		559		1,092		354		302	
P.value	ɑ	F1: p.value: 0.027 Cohen's d: −0.26; F2: p.value: 0.000 Cohen's d: 0.26															
	æ	F1: p.value: 0.000 Cohen's d: −0.71; F2: p.value: 0.017 Cohen's d: 0.20															
	i	F1: p.value: 0.000 Cohen's d: −0.45; F2: p.value: 0.706 Cohen's d: 0.05															
	u	F1: p.value: 0.000 Cohen's d: −0.43; F2: p.value: 0.000 Cohen's d: 1.13															
Male	C. Vowel	ɑ		æ		i		u		ɑ		æ		i		u	
	Formant	F1	F2	F1	F2	F1	F2	F1	F2	F1	F2	F1	F2	F1	F2	F1	F2
	Mean	623	1,314	499	1,765	345	2,206	370	1,522	641	1,318	539	1,819	379	2,082	410	1,385
	SD	148	230	92	205	45	227	57	258	123	260	72	170	121	235	98	277
	#Speakers	6		6		6		6		40		40		40		40	
	#Tokens	78		150		52		42		506		996		329		271	
P.value	ɑ	F1: p.value: 0.234 Cohen's d: −0.14; F2: p.value: 0.890 Cohen's d: −0.01															
	æ	F1: p.value: 0.000 Cohen's d: −0.53; F2: p.value: 0.000 Cohen's d: −0.30															
	i	F1: p.value: 0.000 Cohen's d: −0.29; F2: p.value: 0.000 Cohen's d: 0.53															
	u	F1: p.value: 0.000 Cohen's d: −0.42; F2: p.value: 0.000 Cohen's d: 0.49															

Generally, an anti-formant effect is likely to occur whenever there is a nasal sound after a vowel. The nasal cavity is open when the vowel sound is pronounced, and the velum will go down earlier than expected to prepare the following nasal sound. However, the current corpus does not show that case.

#### 3.2.3 Obstruents

The obstruents are speech sounds that involve a more obstructed vocal tract than the sonorants, resulting in a burst of sound or friction when produced, such as stops, fricatives, and affricates.

##### 3.2.3.1 Obstruent in the onset position

**Figure 7:**
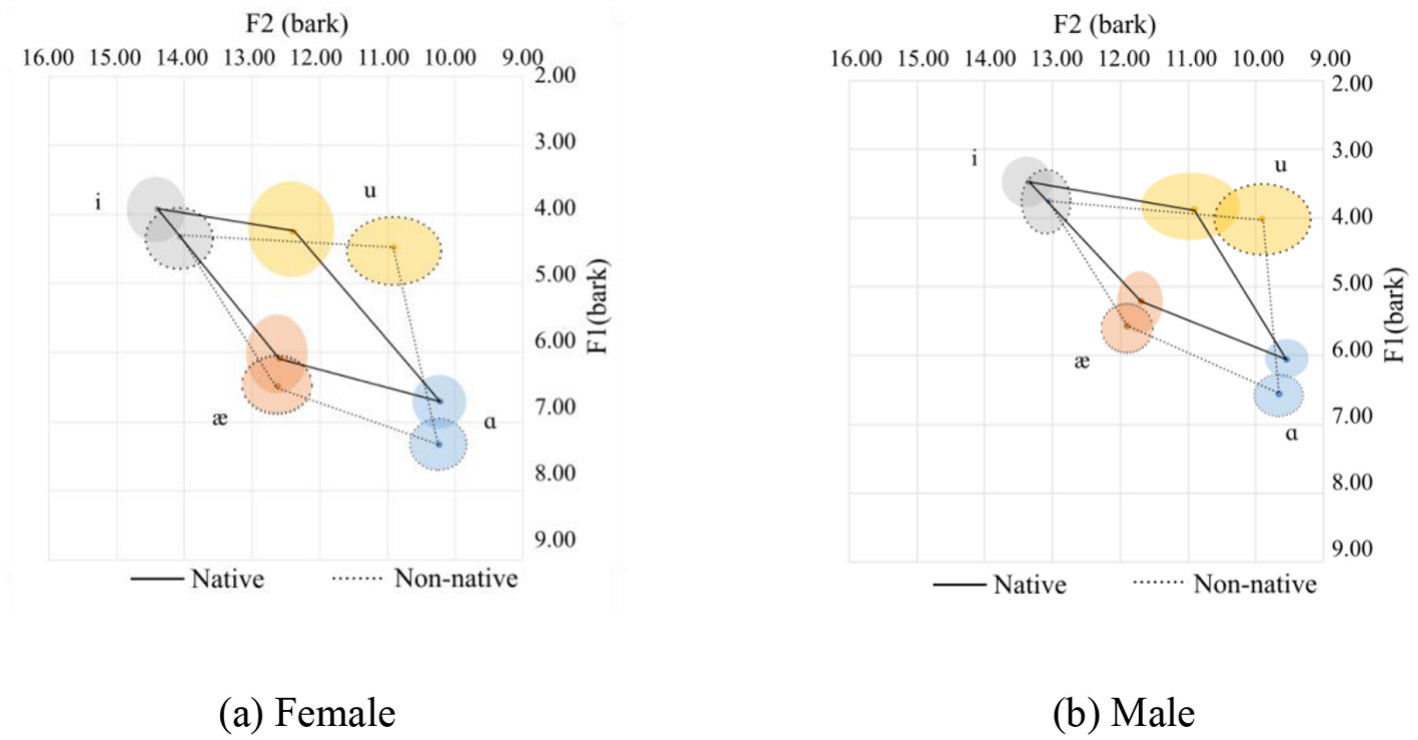
Vowel spaces for onset obstruents. **(a)** Female. **(b)** Male.

In this case, the consonants /t/ /s/ /d/ /z/ /p/ /b/ /v/ /f/ /θ/ /ð*/ /ʃ/ /ʒ/ and /k/ were included. As can be seen from Figure 7a, the vowel space for the Chinese female speakers is significantly larger than that produced by the native female speakers. The Chinese female speakers pronounce the upper corner vowels, /i/ and /u/, with a slightly more posterior tongue position for /i/ and a much more posterior position for /u/. As for the lower corner vowels, these are pronounced with a slightly more frontally and in a lower tongue position than the natives. There is a partial overlap in the pronunciation of all three corner vowels, /ɑ/, /æ/, and /i/, by the native and Chinese speakers and no overlap in the pronunciation of /u/. A similar trend can be observed for the male speakers.

Although the difference between the female and the male speakers can be seen easily in Figures 7a, b, the observed pattern for each is consistent with the general pattern shown in Figure 5. This consonant environment (obstruent in the onset position) does not affect the production of the corner vowels beyond that observed in the general trend in Section 3.1. The statistics are summarized in Table 6 below.

**Table 6:** The F1 and F2 by native and Chinese speakers for onset obstruents.

		**Native**								**Chinese**							
Female	C. Vowel	ɑ		æ		i		u		ɑ		æ		i		u	
	Formant	F1	F2	F1	F2	F1	F2	F1	F2	F1	F2	F1	F2	F1	F2	F1	F2
	Mean	727	1,332	648	1,927	389	2,514	431	1,886	817	1,340	698	1,940	431	2,401	450	1,508
	SD	114	173	155	271	117	277	185	357	113	189	122	289	114	329	139	347
	#Speakers	6		6		6		6		44		44		44		44	
	#Tokens	119		298		373		191		879		2,020		2,583		1,417	
P.value	ɑ	F1: p.value: 0.000 Cohen's d: −0.79; F2: p.value: 0.672 Cohen's d: −0.04															
	æ	F1: p.value: 0.000 Cohen's d: −0.39; F2: p.value: 0.481 Cohen's d: −0.04															
	i	F1: p.value: 0.000 Cohen's d: −0.36; F2: p.value: 0.000 Cohen's d: 0.34															
	u	F1: p.value: 0.087 Cohen's d: −0.13; F2: p.value: 0.000 Cohen's d: 1.08															
Male	C. Vowel	ɑ		æ		i		u		ɑ		æ		i		u	
	Formant	F1	F2	F1	F2	F1	F2	F1	F2	F1	F2	F1	F2	F1	F2	F1	F2
	Mean	637	1,192	534	1,675	339	2,155	384	1,510	705	1,218	577	1,729	369	2,059	400	1,287
	SD	75	127	109	176	88	227	121	342	86	148	98	200	122	220	141	313
	#Speakers	6		6		6		6		40		40		40		40	
	#Tokens	120		302		373		192		795		1,884		2,385		1,283	
P.value	ɑ	F1: p.value: 0.000 Cohen's d: −0.80; F2: p.value: 0.078 Cohen's d: −0.17															
	æ	F1: p.value: 0.000 Cohen's d: −0.43; F2: p.value: 0.000 Cohen's d: −0.27															
	i	F1: p.value: 0.000 Cohen's d: −0.25; F2: p.value: 0.000 Cohen's d: 0.43															
	u	F1: p.value: 0.128 Cohen's d: −0.11; F2: p.value: 0.000 Cohen's d: 0.70															

##### 3.2.3.2 Obstruent in the coda position

**Figure 8:**
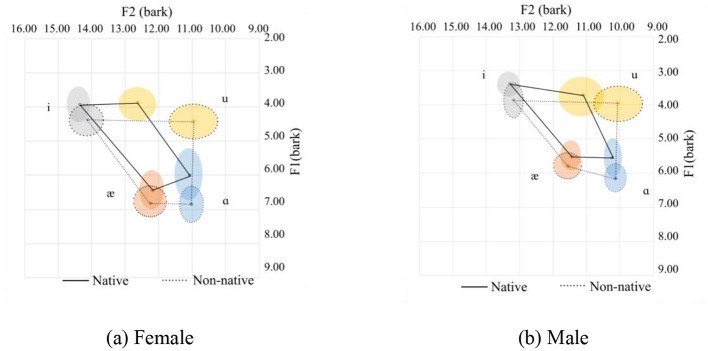
Vowel spaces for coda obstruents. **(a)** Female. **(b)** Male.

In this case, the consonants /t/ /s/ /d/ /z/ /p/ /b/ /v/ /f/ /θ/ /ð*/ /ʃ/ /ʒ/ and /k/ were included. The vowel space for the Chinese female speakers is significantly larger than that produced by the native female speakers due mainly to the pronunciation of /u/. The female Chinese speakers pronounce the upper corner vowels with a lower tongue position. The tongue's elevation is similar for both corner vowels, /i/ and /u/, by the Chinese speakers as opposed to a slightly more elevated tongue position for /u/ by the native female speakers. The tongue position is slightly more posterior for /i/ but is significantly more pulled back for /u/ for pronunciations by the Chinese female speakers. For the lower corner vowels, the native females' tongue position tends to be more elevated but at a similar tongue advancement. In general, the pronunciation of /u/ in the obstruent coda position is more different for the Chinese speakers. These are shown in Figures 8a, b and the statistics are summarized in Table 7.

**Table 7:** The F1 and F2 by native and Chinese speakers for coda obstruents.

		**Native**								**Chinese**							
Female	C. Vowel	ɑ		æ		i		u		ɑ		æ		i		u	
	Formant	F1	F2	F1	F2	F1	F2	F1	F2	F1	F2	F1	F2	F1	F2	F1	F2
	Mean	642	1,526	695	1,803	389	2,497	384	1,945	751	1,509	745	1,829	438	2,428	445	1,518
	SD	194	197	153	200	123	248	120	314	148	169	130	262	115	315	141	345
	#Speakers	6		6		6		6		44		44		44		44	
	#Tokens	88		306		320		163		667		2,228		2,276		1,202	
P.value	ɑ	F1: p.value: 0.000 Cohen's d: −0.70; F2: p.value: 0.386 Cohen's d: 0.09															
	æ	F1: p.value: 0.000 Cohen's d: −0.37; F2: p.value: 0.093 Cohen's d: −0.10															
	i	F1: p.value: 0.000 Cohen's d: −0.42; F2: p.value: 0.000 Cohen's d: 0.22															
	u	F1: p.value: 0.000 Cohen's d: −0.43; F2: p.value: 0.000 Cohen's d: 1.25															
Male	C. Vowel	ɑ		æ		i		u		ɑ		æ		i		u	
	Formant	F1	F2	F1	F2	F1	F2	F1	F2	F1	F2	F1	F2	F1	F2	F1	F2
	Mean	579	1,331	571	1,610	328	2,130	368	1,549	652	1,311	605	1,642	382	2,093	392	1,319
	SD	146	114	106	150	84	208	149	319	108	138	101	209	127	183	140	314
	#Speakers	6		6		6		6		40		40		40		40	
	#Tokens	91		306		337		165		600		2,038		2,075		1,074	
P.value	ɑ	F1: p.value: 0.000 Cohen's d: −0.64; F2: p.value: 0.185 Cohen's d: 0.14															
	æ	F1: p.value: 0.000 Cohen's d: −0.33; F2: p.value: 0.010 Cohen's d: −0.15															
	i	F1: p.value: 0.000 Cohen's d: −0.44; F2: p.value: 0.001 Cohen's d: 0.19															
	u	F1: p.value: 0.041 Cohen's d: −0.16; F2: p.value: 0.000 Cohen's d: 0.73															

Based on the dataset, the obstruent in the coda position does not affect the production of the four corner vowels beyond that observed in Section 3.1 is still applicable.

#### 3.2.4 Different places of articulations

The place of articulation refers to the specific location where speech sounds are produced in the vocal tract. These locations can be divided into two main categories: oral and nasal. The orals include the alveolar, dental, glottal, labials, palatal, post alveolar and velar. Only the alveolar, labials, post alveolar, and velar are considered.

##### 3.2.4.1 Alveolar in the onset position

**Figure 9:**
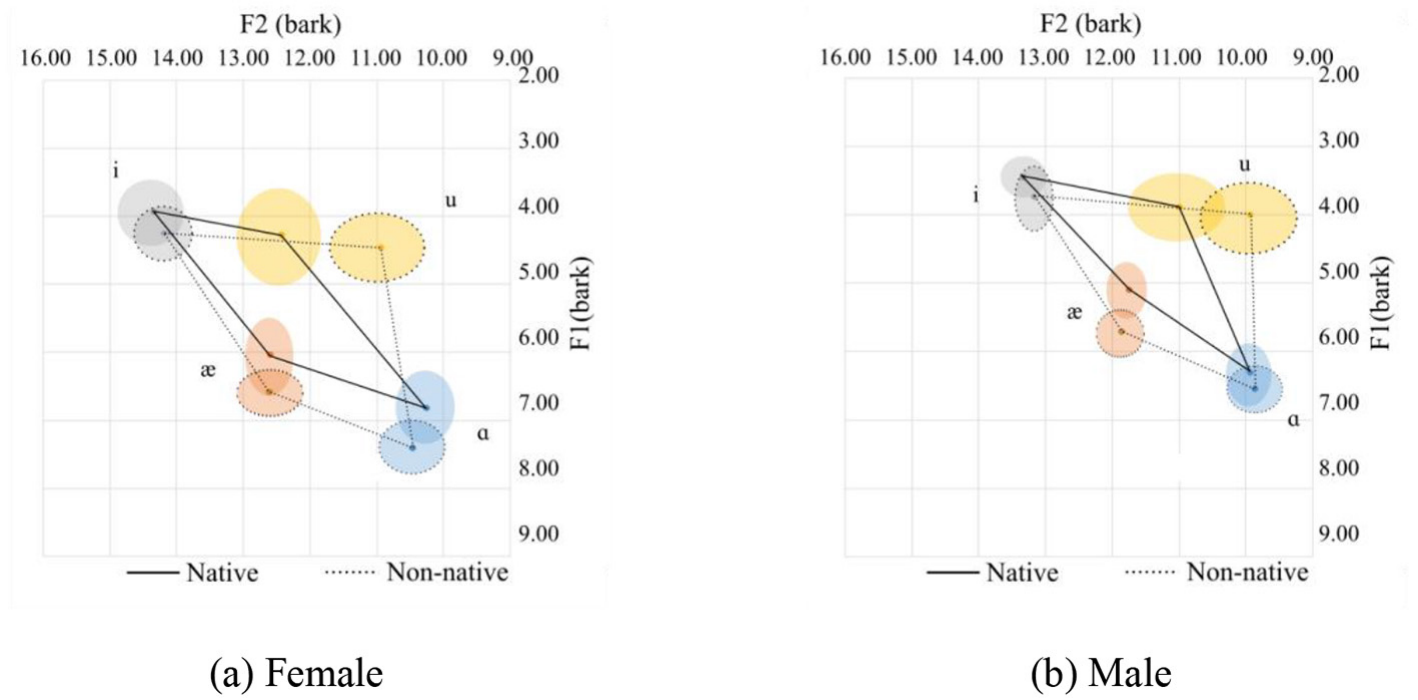
Vowel spaces for onset obstruent alveolars. **(a)** Female. **(b)** Male.

The consonants /t/, /s/, /d/ and /z/ were included in this case. It can be observed from Figures 9a, b that the vowel space for the native speakers is smaller than that of the Chinese speakers. Except for /u/, the sounds produced by the native and Chinese speakers overlap. In general, for the upper corner vowels, the tongue position is more pulled-back and is slightly lower for the Chinese speakers. As for the lower corner vowels, Chinese female speakers pronounce them slightly more frontally and in a lower tongue position. The general trend in Section 3.1 can still be applied to the alveolar sounds in the onset position in this dataset. The formant values are summarized in Table 8 together with their statistics.

**Table 8:** The F1 and F2 by native and Chinese speakers for onset obstruent alveolars.

		**Native**								**Chinese**							
Female	C. Vowel	ɑ	æ	i	u	ɑ	æ	i	u
	Formant	F1	F2	F1	F2	F1	F2	F1	F2	F1	F2	F1	F2	F1	F2	F1	F2
	Mean	748	1,342	641	1,920	389	2,500	434	1,895	829	1,391	710	1,935	423	2,433	449	1,516
	SD	156	199	157	215	119	294	191	358	119	243	102	274	102	288	142	353
	#Speakers	6		6		6		6		44		44		44		44	
	#Tokens	17		101		174		179		125		608		1,275		1,325	
P.value	ɑ	F1: p.value: 0.000 Cohen's d: −0.65; F2: p.value: 0.418 Cohen's d: −0.20															
	æ	F1: p.value: 0.000 Cohen's d: −0.61; F2: p.value: 0.610 Cohen's d: −0.05															
	i	F1: p.value: 0.000 Cohen's d: −0.32; F2: p.value: 0.016 Cohen's d: 0.23															
	u	F1: p.value: 0.215 Cohen's d: −0.10; F2: p.value: 0.000 Cohen's d: 1.07															
Male	C. Vowel	ɑ		æ		i		u		ɑ		æ		i		u	
	Formant	F1	F2	F1	F2	F1	F2	F1	F2	F1	F2	F1	F2	F1	F2	F1	F2
	Mean	673	1,271	520	1,688	335	2,148	385	1,528	705	1,258	594	1,719	369	2,092	399	1,295
	SD	125	143	101	161	69	215	124	342	96	174	100	189	127	182	144	318
	#Speakers	6		6		6		6		40		40		40		40	
	#Tokens	18		104		181		180		115		589		1,193		1,197	
P.value	ɑ	F1: p.value: 0.000 Cohen's d: −0.31; F2: p.value: 0.776 Cohen's d: 0.07															
	æ	F1: p.value: 0.000 Cohen's d: −0.73; F2: p.value: 0.123 Cohen's d: −0.16															
	i	F1: p.value: 0.000 Cohen's d: −0.28; F2: p.value: 0.000 Cohen's d: 0.30															
	u	F1: p.value: 0.232 Cohen's d: −0.09; F2: p.value: 0.000 Cohen's d: 0.72															

##### 3.2.4.2 Alveolar in the coda position

**Figure 10:**
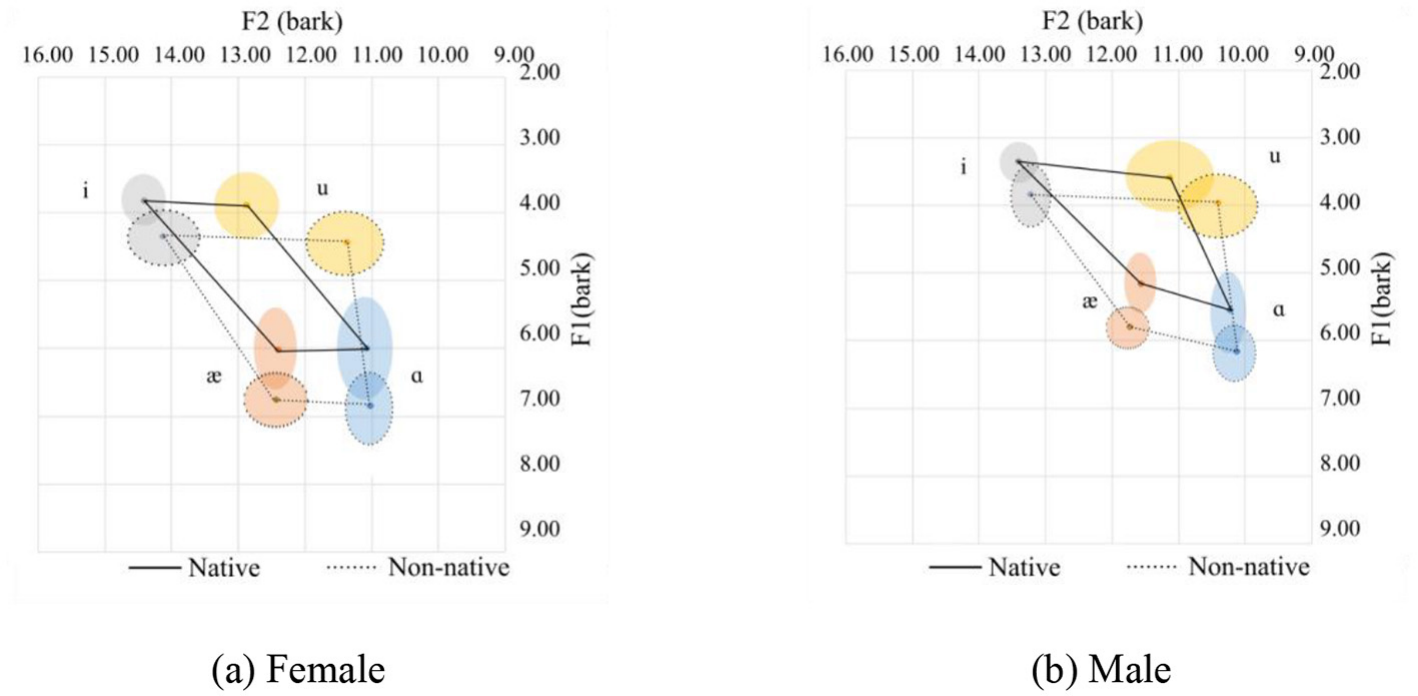
Vowel spaces for coda obstruent alveolars. **(a)** Female. **(b)** Male.

The consonants /t/, /s/, /d/ and /z/ were included. It can be seen from Figure 10 that there is a partial overlap in the sound produced by both groups except for /u/. Besides, the vowel space for the Chinese female speakers is larger. The tongue's elevation is lower for the Chinese females for all corner vowels, and the tongue is more frontal for the lower corner vowels.

Although the difference between females and males can be easily seen in Figures 10a, b, the general pattern of each is consistent with the general pattern shown in Figure 5. Apparently, this consonant environment (Alveolar in the onset position) does not have a significant effect on the production of the corner vowels. The statistics are provided in Table 9 below.

**Table 9:** The F1 and F2 by native and Chinese speakers for coda obstruent alveolars.

		**Native**								**Chinese**							
Female	C. Vowel	ɑ		æ		i		u		ɑ		æ		i		u	
	Formant	F1	F2	F1	F2	F1	F2	F1	F2	F1	F2	F1	F2	F1	F2	F1	F2
	Mean	642	1,526	640	1,864	375	2,516	385	2,010	751	1,509	734	1,879	434	2,430	446	1,607
	SD	194	197	158	183	85	237	121	275	148	169	116	256	100	329	126	296
	#Speakers	6		6		6		6		44		44		44		44	
	#Tokens	88		162		192		59		666		1,176		1,387		431	
P.value	ɑ	F1: p.value: 0.000 Cohen's d: −0.70; F2: p.value: 0.385 Cohen's d: 0.09															
	æ	F1: p.value: 0.000 Cohen's d: −0.77; F2: p.value: 0.456 Cohen's d: −0.06															
	i	F1: p.value: 0.000 Cohen's d: −0.60; F2: p.value: 0.000 Cohen's d: 0.26															
	u	F1: p.value: 0.000 Cohen's d: −0.48; F2: p.value: 0.000 Cohen's d: 1.37															
Male	C. Vowel	ɑ		æ		i		u		ɑ		æ		i		u	
	Formant	F1	F2	F1	F2	F1	F2	F1	F2	F1	F2	F1	F2	F1	F2	F1	F2
	Mean	579	1,331	528	1,639	324	2,167	354	1,555	652	1,311	605	1,687	378	2,107	392	1,385
	SD	146	114	106	122	70	183	143	316	108	138	98	192	112	188	122	272
	#Speakers	6		6		6		6		40		40		40		40	
	#Tokens	91		162		198		59		600		1,081		1,266		393	
P.value	ɑ	F1: p.value: 0.000 Cohen's d: −0.64; F2: p.value: 0.185 Cohen's d: 0.14															
	æ	F1: p.value: 0.000 Cohen's d: −0.77; F2: p.value: 0.000 Cohen's d: −0.26															
	i	F1: p.value: 0.000 Cohen's d: −0.50; F2: p.value: 0.000 Cohen's d: 0.32															
	u	F1: p.value: 0.000 Cohen's d: −0.30; F2: p.value: 0.000 Cohen's d: 0.61															

##### 3.2.4.3 Labials in the onset position

**Figure 11:**
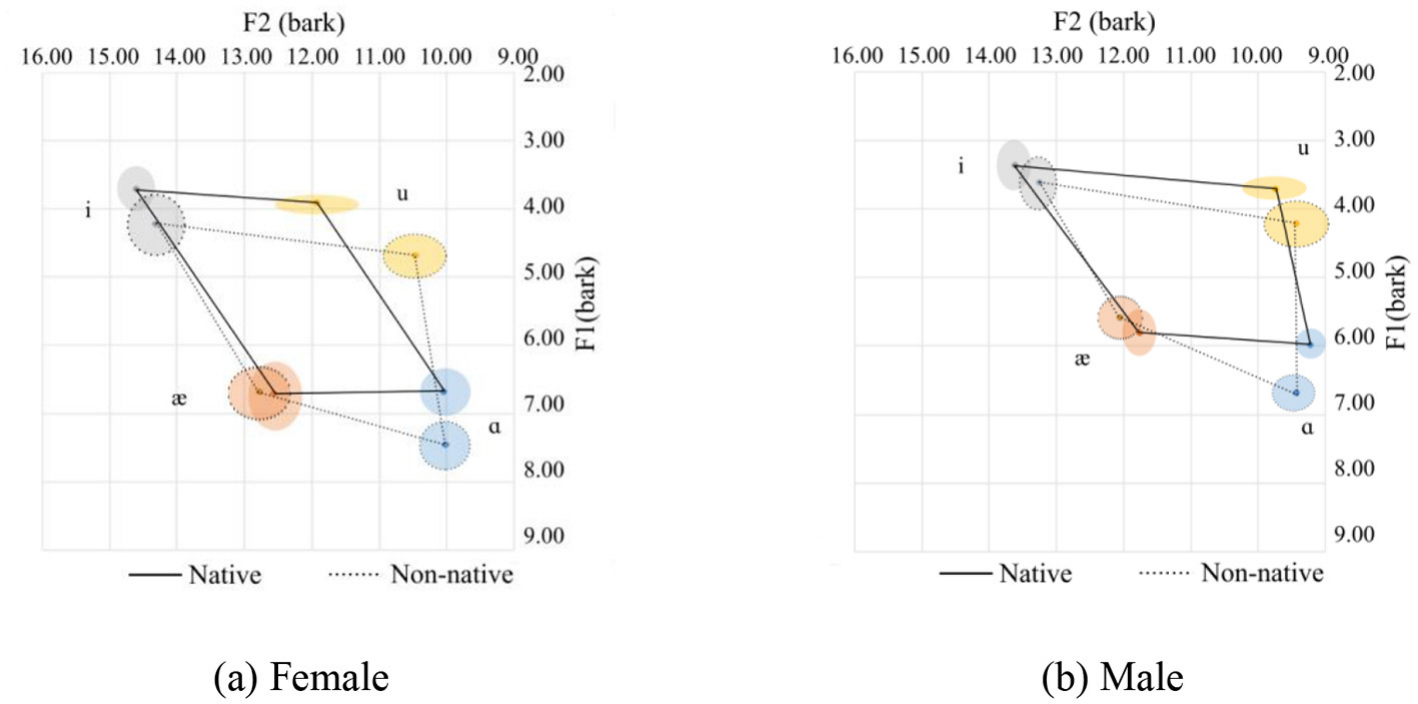
Vowel spaces for onset obstruent labials. **(a)** Female. **(b)** Male.

The consonants /p/, /b/, /v/, and /f/ were included. In general, the vowel space for Chinese female speakers is slightly larger than that of native female speakers as can be seen from Figure 11a. In addition, the sound produced for the two front corner vowels, /i/ and /æ/, is partially overlapped, while the sound produced for the rear corner vowels, /u/ and /ɑ/, is not overlapped. Moreover, it can be readily observed that the difference between the /u/ sound is relatively small for Chinese speakers. Out of the 849 tokens for the vowel /u/ sound, only 24 tokens for the native speakers belong to the labials in the onset position. These tokens belong to the /f/ sound. And all of the 24 /f/ sounds end with the final /d/ sound, which can be traced to the word “food” in the sentence “I like Japanese food, but Korean food is too spicy for me.” Compared with the function words “to” and “you,” “food” is a content word that should be stressed. Consequently, for the female and male native speakers, the /u/ sound has a lower and posterior tongue position which the Chinese speakers also have. The vowel space for the males are given in Figure 11b and the statistics are provided in Table 10.

**Table 10:** The F1 and F2 by native and Chinese speakers for onset obstruent labials.

		**Native**								**Chinese**							
Female	C. Vowel	ɑ		æ		i		u		ɑ		æ		i		u	
	Formant	F1	F2	F1	F2	F1	F2	F1	F2	F1	F2	F1	F2	F1	F2	F1	F2
	Mean	723	1,296	730	1,916	363	2,587	384	1,751	836	1,289	725	1,980	421	2,493	470	1,390
	SD	102	177	146	239	78	217	31	316	107	162	113	254	113	290	83	211
	#Speakers	6		6		6		6		44		44		44		44	
	#Tokens	72		30		109		12		532		204		785		92	
P.value	ɑ	F1: p.value: 0.000 Cohen's d: −1.06; F2: p.value: 0.737 Cohen's d: 0.04															
	æ	F1: p.value: 0.814 Cohen's d: 0.04; F2: p.value: 0.000 Cohen's d: −0.25															
	i	F1: p.value: 0.000 Cohen's d: −0.53; F2: p.value: 0.000 Cohen's d: 0.33															
	u	F1: p.value: 0.000 Cohen's d: −1.08; F2: p.value: 0.000 Cohen's d: 1.60															
Male	C. Vowel	ɑ		æ		i		u		ɑ		æ		i		u	
	Formant	F1	F2	F1	F2	F1	F2	F1	F2	F1	F2	F1	F2	F1	F2	F1	F2
	Mean	628	1,131	606	1,686	328	2,236	362	1,233	721	1,172	577	1,766	354	2,115	418	1,178
	SD	59	82	89	131	91	172	38	193	76	125	85	171	95	175	90	206
	#Speakers	6		6		6		6		40		40		40		40	
	#Tokens	72		30		108		12		480		193		720		85	
P.value	ɑ	F1: p.value: 0.000 Cohen's d: −1.25; F2: p.value: 0.000 Cohen's d: −0.34															
	æ	F1: p.value: 0.000 Cohen's d: 0.33; F2: p.value: 0.000 Cohen's d: −0.48															
	i	F1: p.value: 0.000 Cohen's d: −0.27; F2: p.value: 0.000 Cohen's d: 0.69															
	u	F1: p.value: 0.000 Cohen's d: −0.65; F2: p.value: 0.000 Cohen's d: 0.26															

##### 3.2.4.4 Labials in the coda position

**Figure 12:**
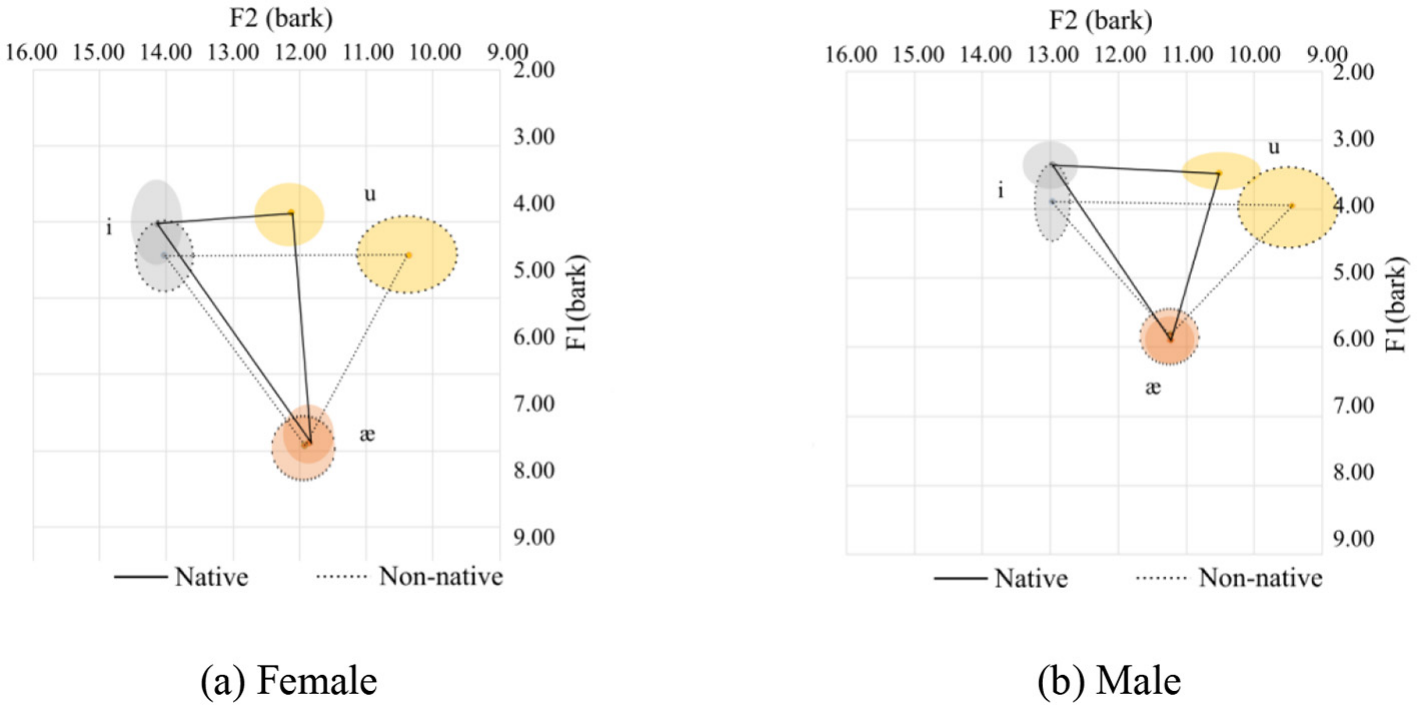
Vowel spaces for coda obstruent labials. **(a)** Female. **(b)** Male.

This section contains only three corner vowels including /i/, /u/, and /æ/, as this dataset does not contain the /ɑ/ sound. This explains why the shape of Figures 12a, b is triangular instead of quadrilateral. In general, the vowel space for the native female speakers is significantly smaller than that of the Chinese female speakers. There is a relatively good overlap for the corner vowels /i/ and /æ/ and no overlap for /u/.

In general, even without the /ɑ/ sound, it can be seen that the pattern of the remaining three corner vowel sounds follows the general trend as in Figure 5. It is worth mentioning that, with obstruent labials in the coda position, it is evident that, for Chinese speakers, the pronunciation of the vowel /æ/ is quite accurate as the centroid of this particular sound is very close to the one for the native speakers regardless of the gender. The summary of the dataset for this section is provided in Table 11.

**Table 11:** The F1 and F2 by native and Chinese speakers for coda obstruent labials.

		**Native**								**Chinese**							
Female	C. Vowel	ɑ		æ		i		u		ɑ		æ		i		u	
	Formant	F1	F2	F1	F2	F1	F2	F1	F2	F1	F2	F1	F2	F1	F2	F1	F2
	Mean	–	–	755	1,715	402	2,420	383	1,796	–	–	762	1,741	446	2,388	448	1,386
	SD	–	–	119	190	147	260	109	265	–	–	147	240	131	274	148	331
	#Speakers	–		6		6		6		–		44		44		44	
	#Tokens	–		126		74		56		–		921		508		396	
P.value	ɑ	F1: p.value: – Cohen's d: –; F2: p.value: – Cohen's d: –															
	æ	F1: p.value: 0.653 Cohen's d: −0.04; F2: p.value: 0.256 Cohen's d: −0.11															
	i	F1: p.value: 0.000 Cohen's d: −0.33; F2: p.value: 0.346 Cohen's d: 0.11															
	u	F1: p.value: 0.000 Cohen's d: −0.45; F2: p.value: 0.000 Cohen's d: 1.26															
Male	C. Vowel	ɑ		æ		i		u		ɑ		æ		i		u	
	Formant	F1	F2	F1	F2	F1	F2	F1	F2	F1	F2	F1	F2	F1	F2	F1	F2
	Mean	–	–	616	1,560	325	2,053	338	1,409	–	–	608	1,563	386	2,029	394	1,196
	SD	–	–	84	172	77	236	66	247	–	–	106	208	144	163	164	313
	#Speakers	–		6		6		6		–		40		40		40	
	#Tokens	–		126		79		54		–		838		465		346	
P.value	ɑ	F1: p.value: –Cohen's d: –; F2: p.value: 0.000 Cohen's d:															
	æ	F1: p.value: 0.435 Cohen's d: 0.07; F2: p.value: 0.852 Cohen's d: −0.01															
	i	F1: p.value: 0.000 Cohen's d: −0.44; F2: p.value: 0.775 Cohen's d: 0.13															
	u	F1: p.value: 0.000 Cohen's d: −0.36; F2: p.value: 0.000 Cohen's d: 0.69															
Female	C. Vowel	ɑ		æ		i		u		ɑ		æ		i		u	
	Formant	F1	F2	F1	F2	F1	F2	F1	F2	F1	F2	F1	F2	F1	F2	F1	F2
	Mean	–	–	755	1,715	402	2,420	383	1,796	–	–	762	1,741	446	2,388	448	1,386
	SD	–	–	119	190	147	260	109	265	–	–	147	240	131	274	148	331
	#Speakers	–		6		6		6		–		44		44		44	
	#Tokens	–		126		74		56		–		921		508		396	
Male	C. Vowel	ɑ		æ		i		u		ɑ		æ		i		u	
	Formant	F1	F2	F1	F2	F1	F2	F1	F2	F1	F2	F1	F2	F1	F2	F1	F2
	Mean	–	–	616	1,560	325	2,053	338	1,409	–	–	608	1,563	386	2,029	394	1,196
	SD	–	–	84	172	77	236	66	247	–	–	106	208	144	163	164	313
	#Speakers	–		6		6		6		–		40		40		40	
	#Tokens	–		126		79		54		–		838		465		346	

##### 3.2.4.5 Post-alveolar in the onset position

The consonants containing /ʒ/ and /ʃ/ are considered for the post-alveolar in the onset position. Only the three corner vowels /ɑ/, /æ/, and /i/ are present.

**Figure 13:**
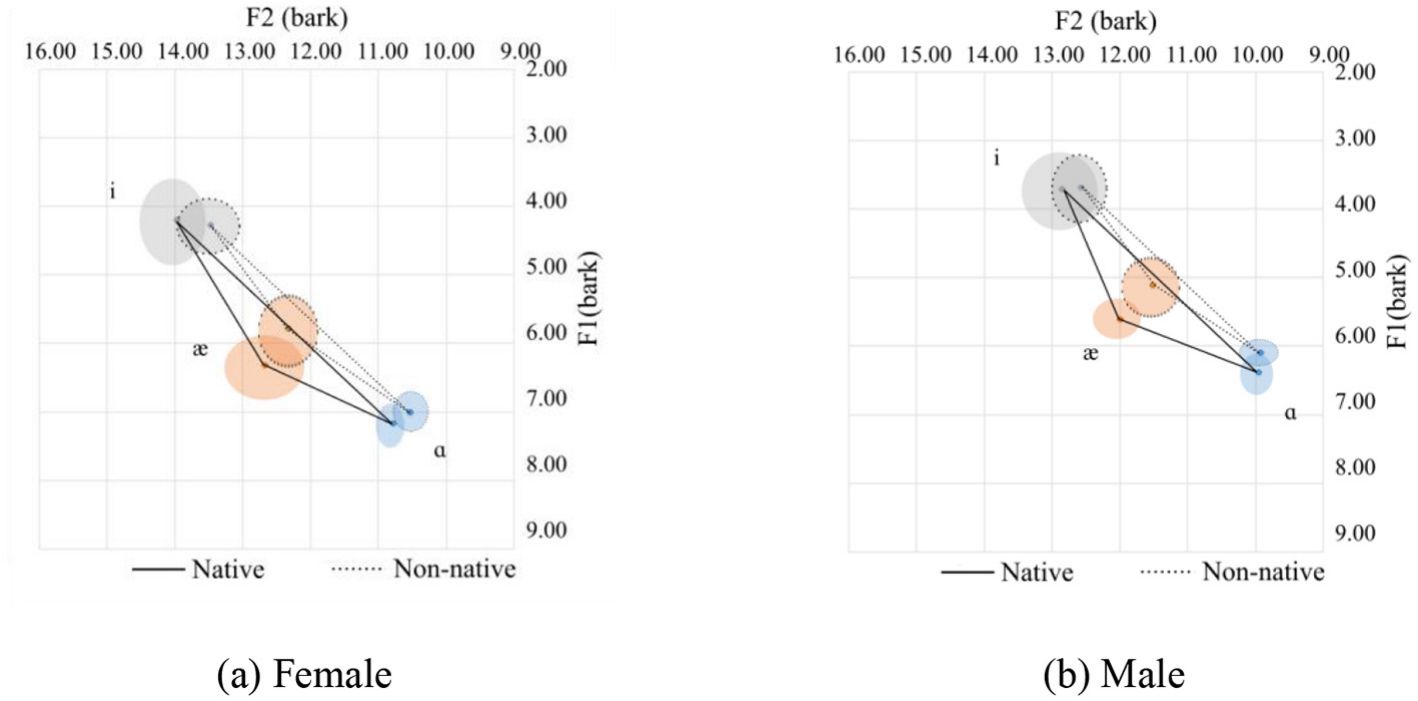
Vowel spaces for onset post-alveolars. **(a)** Female. **(b)** Male.

In general, Chinese speakers pronounce the corner vowels for the post-alveolar in a more pulled-back tongue position. From Figures 13a, b, the height of the tongue is similar for /i/ and higher for both /æ/ and /ɑ/, and the vowel space is smaller for the Chinese speakers. In addition, there is a partial overlap in the pronunciation of all the corner vowels between the two groups. This means that the Chinese speakers' overall pronunciation is quite similar to the native speakers except for the /æ/ sound.

It can be seen that the /æ/ sound for the native speakers is a little lower and more fronted than that of the Chinese speakers. A possible explanation is as follows. Out of the 972 tokens for the /æ/ sound for native speakers, only 72 belong to the /ʒ/ sound in the onset post-alveolar position. Among these 72 tokens, 36 end with the /n/ sound, and the remaining 36 end with the /p/ sound. By looking through the corpus, the /p/ sound belongs to the word “Japanese,” and the /n/ sound belongs to the word “January.” The native speakers acknowledged that these should be pronounced with strength. The first syllable for January or the second syllable for Japanese is consequently stressed. On the contrary, the Chinese speakers did not pronounce it with stress. A summary of the statistics of the formant values are provided in Table 12.

**Table 12:** The F1 and F2 by native and Chinese speakers for onset post-alveolars.

		**Native**								**Chinese**							
Female	C. Vowel	ɑ		æ		i		u		ɑ		æ		i		u	
	Formant	F1	F2	F1	F2	F1	F2	F1	F2	F1	F2	F1	F2	F1	F2	F1	F2
	Mean	788	1,448	678	1,960	423	2,369	–	–	768	1,396	608	1,851	426	2,200	–	–
	SD	95	96	130	346	169	326	–	–	85	118	143	247	106	300	–	–
	#Speakers	6		6		6		–		44		44		44		–	
	#Tokens	6		36		48		–		44		264		353		–	
P.value	ɑ	F1: p.value: 0.000 Cohen's d: 0.23; F2: p.value: 0.000 Cohen's d: 0.44															
	æ	F1: p.value: 0.000 Cohen's d: 0.49; F2: p.value: 0.000 Cohen's d: 0.41															
	i	F1: p.value: 0.847 Cohen's d: −0.02; F2: p.value: 0.000 Cohen's d: 0.55															
	u	F1: p.value: –Cohen's d: –; F2: p.value: –Cohen's d: –															
Male	C. Vowel	ɑ		æ		i		u		ɑ		æ		i		u	
	Formant	F1	F2	F1	F2	F1	F2	F1	F2	F1	F2	F1	F2	F1	F2	F1	F2
	Mean	687	1,448	581	1,753	335	2,148	–	–	767	1,396	594	1,719	369	2,092	–	–
	SD	125	143	101	161	69	215	–	–	96	174	100	189	127	182	–	–
	#Speakers	6		6		6		–		40		40		40		–	
	#Tokens	6		36		181		–		44		476		1,193		–	
P.value	ɑ	F1: p.value: 0.000 Cohen's d: −0.31; F2: p.value: 0.312 Cohen's d: 0.07															
	æ	F1: p.value: 0.000 Cohen's d: −0.73; F2: p.value: 0.002 Cohen's d: −0.16															
	i	F1: p.value: 0.000 Cohen's d: −0.28; F2: p.value: 0.000 Cohen's d: 0.30															
	u	F1: p.value: – Cohen's d: –; F2: p.value: – Cohen's d: –															

##### 3.2.4.6 Velars in the coda position

**Figure 14:**
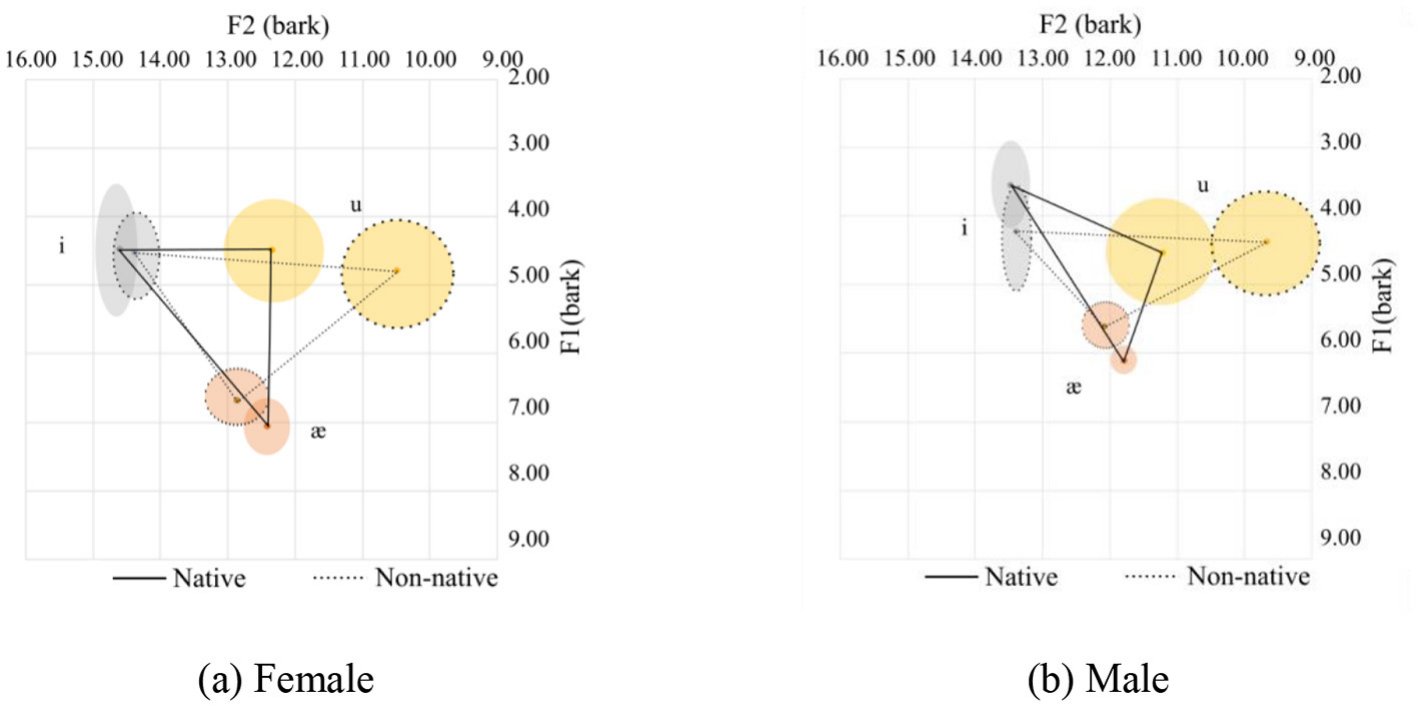
Vowel spaces for coda obstruent velars. **(a)** Female. **(b)** Male.

For the velar in the coda position, there is only the consonant /k/, and the vowel space is composed of only three corner vowels including /æ/, /i/, and /u/. Figures 14a, b together with Table 13 summarizes the data.

**Table 13:** The F1 and F2 by native and Chinese speakers for coda obstruent velars.

		**Native**								**Chinese**							
Female	C. Vowel	ɑ		æ		i		u		ɑ		æ		i		u	
	Formant	F1	F2	F1	F2	F1	F2	F1	F2	F1	F2	F1	F2	F1	F2	F1	F2
	Mean	–	–	778	1,870	375	2,587	385	1,874	–	–	742	2,004	433	2,504	445	1,422
	SD	–	–	121	189	235	235	180	388	–	–	116	257	155	234	242	391
	#Speakers	–		6		6		6		–		44		44		44	
	#Tokens	–		18		193		18		–		130		210		123	
P.value	ɑ	F1: p.value: –Cohen's d: –; F2: p.value: – Cohen's d: –															
	æ	F1: p.value: 0.000 Cohen's d: 0.30; F2: p.value: 0.000 Cohen's d: −0.53															
	i	F1: p.value: 0.000 Cohen's d: 0.02; F2: p.value: 0.000 Cohen's d: 0.35															
	u	F1: p.value: 0.001 Cohen's d: −0.17; F2: p.value: 0.000 Cohen's d: 1.15															
Male	C. Vowel	ɑ		æ		i		u		ɑ		æ		i		u	
	Formant	F1	F2	F1	F2	F1	F2	F1	F2	F1	F2	F1	F2	F1	F2	F1	F2
	Mean	–	–	643	1,697	350	1,697	353	1,588	–	–	582	1,775	428	2,155	392	1,245
	SD	–	–	55	103	144	181	182	383	–	–	89	181	188	146	203	335
	#Speakers	–		6		6		6		–		40		40		40	
	#Tokens	–		18		30		18		–		119		188		155	
P.value	ɑ	F1: p.value: – Cohen's d: –; F2: p.value: – Cohen's d: –															
	æ	F1: p.value: 0.000 Cohen's d: 0.71; F2: p.value: 0.000 Cohen's d: −0.45															
	i	F1: p.value: 0.000 Cohen's d: −0.42; F2: p.value: 0.000 Cohen's d: −3.02															
	u	F1: p.value: 0.028 Cohen's d: 0.07; F2: p.value: 0.000 Cohen's d: 1.00															

In general, the vowel space for Chinese female speakers is significantly larger than that of the native females owing to the pronunciation of /u/. Of the 849 tokens produced by the native speakers for the /u/ sound, 36 tokens end with the velar /k/ sound. Out of these, 12 tokens are for the word “you,” 12 tokens are for the word “to,” and the remaining 12 tokens are for the word “who.” The words “you,” “to,” and “who” server as the functional words in the sentence, which explains why the /u/ sound is pronounced with reduced emphasis like the schwa. In contrast, Chinese speakers do not consider these as functional words and pronounce them with emphasis.

### 3.3 Discussion

#### 3.3.1 Vowel space

**Figure 15:**
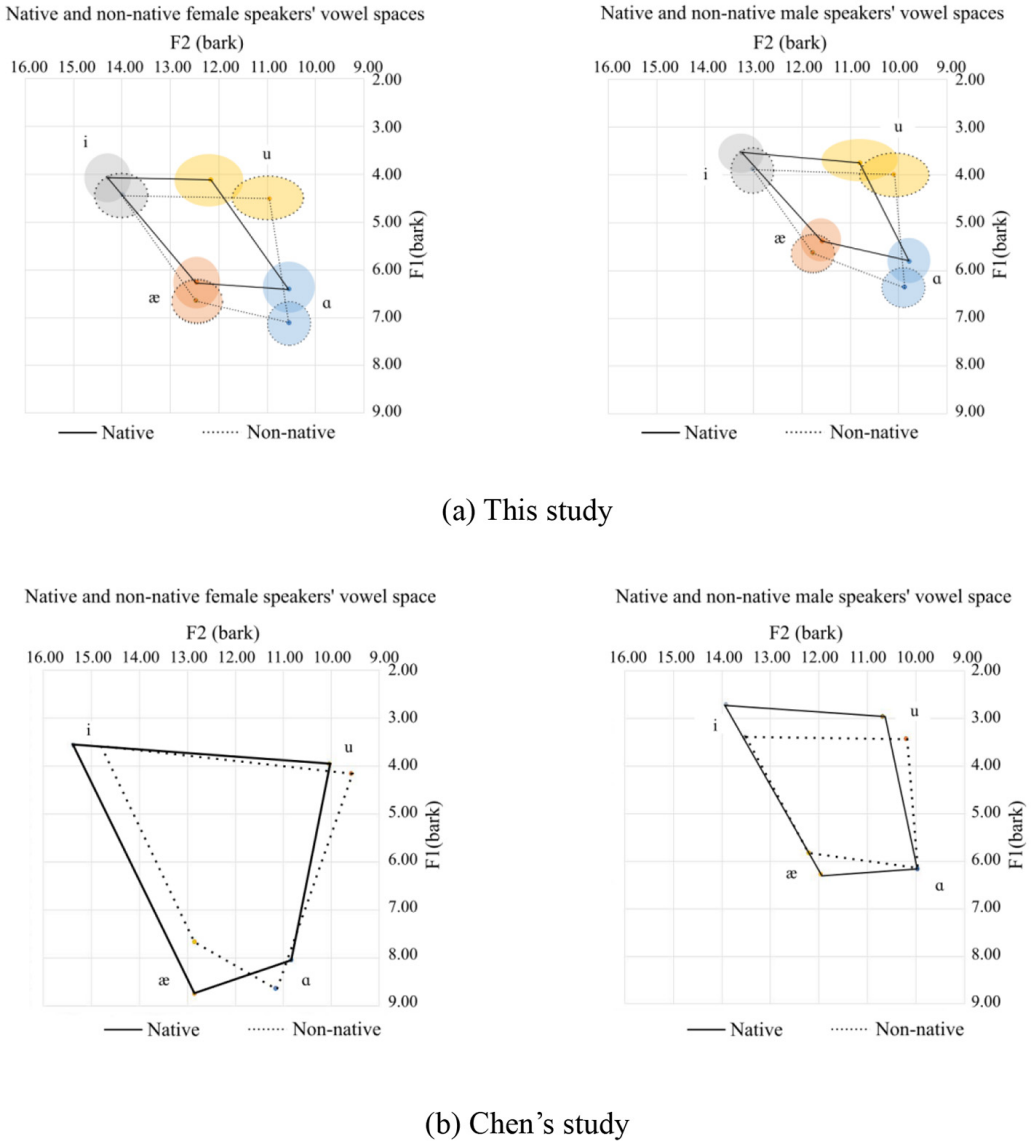
Comparison of vowel spaces between this study and Chen's study. **(a)** This study. **(b)** Chen's study.

It's not difficult to see that compared to Chen's data, the vowel space derived from this corpus is much smaller, regardless of whether the speakers are native or Chinese, male or female. One possible explanation for this is that the speech samples for the present study are mostly from a sentence instead of an isolated word, that is to say, vowels in this study are from “continuous speech” with numerous segments that exercise the full articulation of the tongue in the oral cavity (Lindblom, 1983). In this case, it is highly probable that vowels are produced without being fully articulated, and the overall speakers' vowel space should shrink accordingly. In contrast, the vowels from Chen's study were read in the same carrier sentence: “Say _____ again five times.” In this case, the subjects put some stress on the target word, resulting in the vowels included in the target words being produced in a fully articulated form. The vowel space from the two studies are provided in Figures 15a, b.

#### 3.3.2 Effect of the consonant context on the vowel production

The vowels in this study are not from a uniform carrier sentence as carried out in Chen (1999), Peterson and Barney (1952), and Hillenbrand et al. (1995). Although the same vowels are pooled to calculate the mean and standard deviation of the vowel, words, including the target vowel, are different. This means that the same vowels are not from a homogenous phonological environment. Segmental variations may cause changes in the formant values because of coarticulation. The general trend shown in Figure 5 may have different facets. Instead of dividing segmental environments according to the identity of onset and coda segments, we grouped some consonants according to their phonological features, such as sonorant, obstruent, labial, etc. Compared to the general trend in Figure 5, not all phonological features have a conspicuous effect, as the dataset did not contain enough tokens. The groups are summarized in Table 14.

**Table 14:** Summary of the contextual consonantal effects on the vowels.

**Corner vowel**	**Context in a syllable**	**Affected by**	**Effect**
/i/	Sonorant coda (/r/^*^,/l/^*^,/j/^*^,/w/)	With the/w/sound after/i/sound	F1 is slightly lower, F2 is significantly lower
/u/	Labials Onset (/p/^*^,/b/^*^,/v/^*^,/f/)	Mainly from the stressed content word “*food*”	F2 is significantly lower
Velar Coda (/k/)	Mainly from the unstressed content word “*you*,” “*to*,” and “*who*”	F2 is significantly higher compared with the general trend	
/æ/	Nasal Coda (/n/,/m/^*^,//η//^*^)	With the/n/sound after/æ/sound	F1 is significantly lower
Post-alveolar onset (/ʒ/,/ʃ/^*^)	Words are “*Japanese*”/”*January*”	F1 is significantly higher observed particularly among the Chinese speakers
/ɑ/	Sonorant onset (/r/^*^,/l/^*^,/j/^*^,/w/)	With the /w/ sound before /ɑ/ sound	F1 is significantly lower/F2 is slightly higher	

These observations and their explanations are as follows:

- The labial-velar sound /w/ in the coda position mainly affects the /i/ corner vowel. This /w/ sound requires the rounding of the lips and raising of the tongue, resulting in /i/ being pronounced similarly to a velar. When /w/ precedes /i/, the lips anticipate a rounded position, while the tongue is relatively pulled back, resulting in a lower F1 and F2 value.
- The /u/ corner vowel is affected by an /f/ consonant in the labial coda position. The dataset contains only 24 tokens for the /u/ vowel for the native speakers belonging to the /f/ sound, which is a labial in the onset position. And all of the 24 F sounds end with the final /d/ sound, which can be traced to the word “food” in the sentence “I like Japanese food, but Korean food is too spicy for me.” Compared with the function words “to” and “you,” “food” is a content word that should be stressed. Consequently, the native speakers produce the /u/ sound with a lower and more pulled-back tongue position than the Chinese speakers.

/u/ is also affected by the consonant velar /k/ in the coda position due to the words “you,” “to,” and “who,” which serve as the function word in the sentences. This explains why the /u/ sound is pronounced with reduced emphasis, like the schwa by the native speakers. In contrast, Chinese speakers do not consider these as functional words and pronounce them with emphasis.

- The /æ/ corner vowel was mostly affected by the anticipation of the nasal /n/ sound, which requires the tongue to be raised to produce the alveolar nasal sound. Consequently, this anticipation moves the tongue closer to the alveolar ridge area, which naturally lowers the F1 value.

It can be observed that the /æ/ sound for the native speakers is slightly lower and more fronted than that of the Chinese speakers. A possible explanation is that the influence is due to the /ʒ/ sound in the onset post-alveolar position followed by either an /n/ or a /p/ sound. By looking through the corpus, the one with the /p/ sound belongs to the word “Japanese,” and the one with the /n/ sound belongs to the word “January.” The native speakers know that these should be pronounced with a strong tone, regardless of whether the first syllable is for January or the second for Japanese. On the contrary, the Chinese speakers did not pronounce it with a relatively strong tone.

- The /ɑ/ corner vowel can be affected by the /w/ sound in the onset position. /w/ is a labial-velar sound, which requires the rounding of the lips and raising the tongue, similar to the pronunciation of the velars. When /w/ precedes /ɑ/, which is a low vowel, the lips tend to remain rounded with the tongue still in the velar position resulting in a more rounded or protruded lips position for the /ɑ/ sound. This lowers the F1 and F2 values.

## 4 Conclusions

In this contribution, the pronunciation of the four corner vowels /i/, /u/, /æ/, and /ɑ/ from the AESOP-ILAS dataset source has been studied to understand better the factors that can help Chinese speakers achieve more native like pronunciation. Unlike previous studies, which measured the formants at the middle of the target vowel, the formants in this study were measured at the start, middle, and end portions of the vowel pronunciation, and then their averages were taken. A significant reduction in the vowel space was observed in this study compared to previous studies, irrespective of the speaker's linguistic background or gender. This observation could be attributed to the methodology used to collect the speech samples as the vowel sounds were derived from a predominantly sentence-based corpus, meaning they were extracted from continuous speech where the tongue fully exercises its articulatory potential. Consequently, the vowels produced were not fully articulated, resulting in a reduced vowel space.

Unexpectedly, the vowel space for the Chinese speakers was larger than that of the native speakers compared with previous studies due to the speech samples coming from a sentence rather than an isolated word, which painted a more reliable picture of the vowel space. In addition, Chinese speakers pronounced the corner vowels with lower F1 and F2 values or with a lower and pulled-back tongue position, and the most striking difference was with the pronunciation of /ɑ/ and /u/.

The differences in the /u/ corner vowel were traced back to the pronunciation of “to,” “you,” and “who,” which significantly raised F2 for /u/ in the velar coda position with consonant /k/, and to the word “food” in the labial onset position, which significantly lowered F2. As for /ɑ/, it was affected by a preceding /w/ sound in the sonorant onset position, which lowered F1 and raised F2. The difference in /i/ was mostly observed in the sonorant coda position with a following /w/ sound, which lowered F1 and F2. /æ/ was mostly affected in the nasal coda position by a preceding /n/ sound which lowered the F1 formant, and in the post-alveolar in the onset position due to the pronunciation of the words “January” and “Japanese,” which raised F1. It is believed that by incorporating when to stress a certain word in a sentence, the pronunciation of the Chinese speakers will be greatly improved.

## Data Availability

The original contributions presented in the study are included in the article/supplementary material, further inquiries can be directed to the corresponding author.
