# Supplementary material for: Effect of the consonant context on the corner vowel produced by native and Chinese speakers: based on AESOP corpora
Source: Front Psychol. 2025 Sep 17;16:1598904. doi: 10.3389/fpsyg.2025.1598904 (PMC12483891; doi:10.3389/fpsyg.2025.1598904)
Supplement: Supplementary file 1 [file Supplementary_file_1.docx]

Appendix A

| Record Number | Sentence |
| --- | --- |
| 1000 | I said apartment five times. |
| 1001* | I said overnight five times |
| 1002 | I said misunderstand five times. |
| 1003 | I said supermarket five times. |
| 1004 | I said money ten times. |
| 1005 | I said hospital ten times. |
| 1006* | I said white wine ten times . |
| 1007* | I said elevator ten times. |
| 1008* | I said available ten times. |
| 1009* | I said information ten times. |
| 1010 | I said January ten times. |
| 1011 | I said experience ten times. |
| 1012 | I said California ten times. |
| 1013 | I said Vietnamese ten times . |
| 1014 | I say department store ten times. |
| 1015* | I said morning ten times . |
| 1016 | I said video ten times. |
| 1017 | I say tomorrow ten times. |
| 1018 | I said Japanese ten times. |
| 1019 | I said afternoon ten times. |

The asterisk (*) indicates that there are no corner vowels in the sentence.

Appendix B

| Record Number | Sentence |
| --- | --- |
| 2000 | Do you need any money? |
| 2001 | Did he go to the hospital? |
| 2002 | Has jane found an apartment |
| 2003 | Can packages be shipped overnight? |
| 2004 | Would you like a glass of white wine |
| 2005 | Where is the elevator? |
| 2006 | When will bill be available? |
| 2007 | Who can give me the information? |
| 2008 | Why do you always misunderstand? |
| 2009 | Where is the nearest supermarket? |
| 2010 | In December and January, the sun rises at seven in the morning. |
| 2011 | Although Fred didn't have any experience, he had no trouble learning how to make a video. |
| 2012 | When sue left this evening for California, she said she would call me tomorrow. |
| 2013 | If you want to learn Vietnamese, I think it will be easier than Japanese. |
| 2014 | If you want to check out the new department store, we can go this afternoon. |

Appendix C

| Record Number | Sentence |
| --- | --- |
| 3000 | No. His **MONEY** was taken, but they didn’t take his computer. |
| 3001 | No. You should go to a **HOSPITAL** for blood tests. |
| 3002 | No. this is an **APARTMENT** building, not a commercial building. |
| 3003 | No. we need **OVERNIGHT** delivery. |
| 3004 | No. I ordered **WHITE** **WINE**, not coke. |
| 3005 | You should take the **ELEVATOR** instead of the stairs. |
| 3006 | Someone is already sitting there. Are there any **AVAILABLE** tables by the window? |
| 3007 | You should have gone to the **INFORMATION** desk. |
| 3008 | I didn't **MISUNDERSTAND** the question ; i just chose not to answer it. |
| 3009 | No. I usually buy fruit at the **SUPERMARKET** because they stay open later. |
| 3010 | No. But I think **EXPERIENCE** is more important than training. |
| 3011 | No. they speak **VIETNAMESE** in Vietnam . |
| 3012 | No. Teresa lives in **CALIFORNIA** now. |
| 3013 | No. it's in **JANUARY** this year |
| 3014 | No. Mary is taking a **MORNING** flight. |
| 3015 | No. **VIDEO** recordings are not allowed. |
| 3016 | No. Anna is leaving **TOMORROW** for Chicago. |
| 3017 | I like **JAPANESE** food , but Korean food is too spicy for me. |
| 3018 | I would prefer to meet in the **AFTERNOON** instead. |
| 3019 | No , I work at a **DEPARTMENT** store. |

The boldface words were requested to be read with an emphasis.

Appendix D

| Record Number | Sentence |
| --- | --- |
| 4000 | If the birthday party wasn't for Mary, then who was it for? |
| 4001 | Jane saw a picture of the boy she was fond of. |
| 4002 | John went to visit the woman he had written to. |
| 4003 | I can run faster than you can. |
| 4004 | He went to a fancy dress party as a guest , but what did he dress as? |

Appendix E

| Record Number | Sentence |
| --- | --- |
| 5000 | When Alice leaves, **TOM** will be upset. |
| 5001 | When Alice leaves Tom, **WE'LL** be upset. |
| 5002 | **I’LL** look after the children until lunch time. |
| 5003 | I’ll **LOOK AFTER** the children have left. |
| 5004 | The fight is over, **MARY**. |
| 5005 | The fight is **OVER**, Mary. |
| 5006 | He's a good boy, **ISN'T** he? |
| 5007 | **HE'S** a good boy, isn't he? |
| 5008 | She knows everyone, **DOESN'T** she? |
| 5009 | She knows **EVERYONE**, doesn't she? |

Appendix F

| Record Number | Sentence |
| --- | --- |
| 6000 | The north wind and the sun were disputing which was the stronger when a traveler came along wrapped in a warm cloak. they agreed that the one who first succeeded in making the traveler take his cloak off should be considered stronger than the other.  Then the north wind blew as hard as he could, but the more he blew the more closely did the traveler fold his cloak around him; and at last the north wind gave up the attempt.  Then the sun shone out warmly, and immediately the traveler took off his cloak. And so the north wind was obliged to confess that the sun was the stronger of the two. |

Appendix G

| Record Number | Sentence |
| --- | --- |
| 7000 | When would you like to travel? |
| 7001 | Did you say the twenty second or the twenty seventh? |
| 7002 | Would you like a window seat or an aisle seat? |
| 7003 | Would you like a special dinner? |
| 7004 | When would you like to reserve your returning flight? |
| 7005 | Your flight, B R three one seven, will depart from C K S airport at eleven fifteen A M on November twenty second. you will arrive at Narita airport at two fifty pm. you will transfer to flight eight O nine to New York J F K airport which departs at seven O eight P M from gate thirteen f. you will land at J F K airport at four thirty P M on November twenty second. |
| 7006 | The flight leaves from gate thirteen F, not gate thirty F. |
| 7007 | May I have your name? |
| 7008 | Is that L U C Y H A S E G A W A J O H N S O N? |
| 7009 | May I have your credit card number? |
| 7010 | So that's visa five nine two four eight zero one three six seven zero two three five one six expiration date nine two thousand twelve. |
| 7011 | May I have your billing address? |
| 7012 | Let me repeat that back to you fourteen twenty-five lakeshore drive, apartment forty-seven B, Chicago, Illinois six O one night five. |
| 7013 | May I have your contact phone number? |
| 7014 | Is that six O nine four seven two one three five eight? |
| 7015 | Is there anything else I can help you with this morning? |
| 7016 | Goodbye and thank you for calling Eva airlines. |
